# Membrane omega-3 fatty acids modulate the oligomerisation kinetics of adenosine A_2A_ and dopamine D_2_ receptors

**DOI:** 10.1038/srep19839

**Published:** 2016-01-22

**Authors:** Ramon Guixà-González, Matti Javanainen, Maricel Gómez-Soler, Begoña Cordobilla, Joan Carles Domingo, Ferran Sanz, Manuel Pastor, Francisco Ciruela, Hector Martinez-Seara, Jana Selent

**Affiliations:** 1 Research Programme on Biomedical Informatics (GRIB), Department of Experimental and Health Sciences Universitat Pompeu Fabra, IMIM (Hospital del Mar Medical Research Institute), Barcelona, Spain; 2 Department of Physics, Tampere University of Technology, Tampere, Finland; 3 Facultat de Medicina, IDIBELL, Universitat de Barcelona, Barcelona, Spain; 4 Facultat de Biologia, Universitat de Barcelona, Barcelona, Spain; 5 Faculty of Sciences, University of Ghent, Gent, Belgium

## Abstract

Membrane levels of docosahexaenoic acid (DHA), an essential omega-3 polyunsaturated fatty acid (*ω*-3 PUFA), are decreased in common neuropsychiatric disorders. DHA modulates key cell membrane properties like fluidity, thereby affecting the behaviour of transmembrane proteins like G protein-coupled receptors (GPCRs). These receptors, which have special relevance for major neuropsychiatric disorders have recently been shown to form dimers or higher order oligomers, and evidence suggests that DHA levels affect GPCR function by modulating oligomerisation. In this study, we assessed the effect of membrane DHA content on the formation of a class of protein complexes with particular relevance for brain disease: adenosine A_2A_ and dopamine D_2_ receptor oligomers. Using extensive multiscale computer modelling, we find a marked propensity of DHA for interaction with both A_2A_ and D_2_ receptors, which leads to an increased rate of receptor oligomerisation. Bioluminescence resonance energy transfer (BRET) experiments performed on living cells suggest that this DHA effect on the oligomerisation of A_2A_ and D_2_ receptors is purely kinetic. This work reveals for the first time that membrane *ω*-3 PUFAs play a key role in GPCR oligomerisation kinetics, which may have important implications for neuropsychiatric conditions like schizophrenia or Parkinson’s disease.

Several studies have found substantially lower levels of docosahexaenoic acid (DHA) in the brains of individuals with mental^1^ or neurological disorders^2^,^3^. DHA is an omega-3 polyunsaturated fatty acid (*ω*-3 PUFA) of 22 carbons and 6 double bonds (22:6n3) that has been shown to be essential for the development^4^ and maintenance of adequate brain function^5^,^6^. The high levels of DHA found in specialized cell platforms such as retinal rod outer segments or neuronal cells seem to provide these membranes with particular biophysical properties such as an increased lipid mobility and amenability to deformation^7^,^8^. Furthermore, DHA is able to affect the lateral organization of cell membranes through the formation of the so-called anti-raft domains which promote the function of specific proteins^9^. The unique biophysical properties of DHA along with its potential neuroprotective effects^5^,^10^ have made DHA a promising candidate against certain neurodegenerative disorders^11^,^12^.

In addition, recent studies have shown that the lipid composition of cell membranes can modulate the function of key membrane proteins such as G protein-coupled receptors (GPCRs)^13^,^14^. GPCRs are involved in a wide range of diseases and are particularly important for several major psychiatric disorders^15^. Therefore, understanding the role of the membrane environment on the dynamics and function of GPCRs has become a research priority in this field. For instance, the high amount of DHA present in retinal rod cell membranes is known to modulate the function of rhodopsin, a widely studied GPCR specific to these cells, by increasing lateral diffusion and thus the efficiency of G-protein coupling^16^. The modulatory effect of DHA on rhodopsin was first described by Mitchell *et al.*^17^ and has since been further studied using experimental^16^,^18^ and computational^19^,^20^ methods. DHA is therefore known to influence the biology of rhodopsin and could potentially modulate other GPCRs in other DHA-enriched tissues such as the brain^4^.

However, an additional level of complexity adds to the overall picture of DHA–GPCR modulation: GPCRs have recently been found to function as dimers or higher order oligomers, and despite initial controversy, the existence and relevance of GPCR oligomerisation has gained broad acceptance^21^. Interestingly, impaired crosstalk between specific GPCR heteromers seems to affect GPCR signalling resulting in defective neurotransmission and brain dysfunction^22^,^23^. Therefore, GPCR heteromers are nowadays desired drug targets^24^,^25^ and have inspired new drug strategies such as the use of heterobivalent or dual acting ligands^26^,^27^. In particular, the study of adenosine A_2A_ and dopamine D_2_ receptors, which have been shown to form dimers^28^,^29^ and oligomers^30^,^31^ is becoming highly relevant in neuropsychiatry^32^,^33^. A specific balance of membrane A_2A_ and D_2_ oligomers is thought to be behind the altered signalling cascade observed in Parkinson’s disease and schizophrenia^34^,^35^.

Given the importance of GPCR oligomerisation and membrane DHA in brain dysfunction, the question that naturally arises is do membrane lipids affect GPCR oligomerisation? It seems possible that this is the mechanism through which DHA modulates GPCR biology, and subsequently neurological disease processes. In this study we compared the effects of high and low levels of DHA on the homo- and heteroligomerisation of A_2A_ and D_2_ receptors. We performed molecular dynamics (MD) simulations of the self-assembly process of A_2A_ and D_2_ receptors simultaneously embedded in multicomponent model membranes reaching an exceptionally long total simulation time of nearly 4 ms. We then compared the effect of high and low levels of membrane DHA on protein aggregation and studied the particular affinity between this lipid and A_2A_ and D_2_ receptors. We complemented MD simulations with bioluminescence resonance energy transfer (BRET) experiments in living cells in order to study the effect of DHA on the degree of oligomerisation in the steady state.

Receptor oligomerisation is a dynamic process comprising receptor association and dissociation events. Unfortunately, no single experimental approach can capture this complex process combining high-resolution conditions at the required time scale. Therefore, in this paper we focus on the molecular details behind fast events of receptor oligomerisation, namely receptor association (microsecond time scale), using all-atom and coarse-grained MD simulations. In addition, we use BRET experiments in living cells to explore receptor association/dissociation events at longer time scales needed to achieve equilibrium (millisecond time scale). Our results suggest for the first time that GPCR oligomerisation kinetics can be modulated by membrane *ω*-3 PUFAs. The concentration of DHA present in the healthy brain provides membranes with fluidity required for rapid receptor diffusion. Additionally, DHA seems to foster oligomerisation by promoting membrane phase separation. BRET experiments do not, however, find differences in the oligomerisation state between DHA-enriched and DHA-depleted membranes in the steady state suggesting that the effect of DHA on protein oligomerisation is mainly a kinetic one. These results provide a molecular link between membrane lipid composition and the rate of GPCR oligomerisation, which could help in the development of new strategies to treat major neurological disorders.

## Methods

### Coarse-grained MD simulations

We performed a complete set of coarse-grained (CG) MD simulations to study the oligomerisation of A_2A_ and D_2_ receptors in membranes of various compositions using the Martini force field^36^,^37^. First, a small patch (i.e. approximately 11 × 11 nm^2^ in the membrane plane) containing two receptors separated from each other (i.e. distance between the positions of their centres of mass (COM) > 9 nm) was created. CG models of A_2A_ and dopamine D_2_ receptors were built based on the inactive crystal structures PDB:3EML and PDB:3PBL, respectively. The native sequence of these structures was used. Unresolved residues were added and the intracellular loop 3 was omitted. Subsequently, the content of this patch was independently replicated to create larger systems. In order to study protein aggregation, we considered two receptors to be in direct contact if the distance between the positions of their COMs was <4.2 nm. Lipid-mediated contacts are also accounted for, as long as the cut-off distance criteria is met. Detailed information of each system and thorough building, simulation and analysis protocols can be found in the Supporting Information (SI). Initially, we simulated nine A_2A_ and nine D_2_ receptors embedded^38^ in two model membranes of different lipid composition. These compositions aimed to reflect the general brain lipid profiles previously observed in post-mortem studies of healthy and diseased individuals^1^,^2^,^3^. Briefly, the healthy-like (rich in DHA) and diseased-like (poor in DHA) models contained 21% and 6% of a DHA-phospholipid (i.e. SDPC, 1-stearoyl-2-docosahexaenoyl-*sn*-glycero-3-phosphocholine), respectively; the diseased-like model was compensated with a higher fraction of saturated lipids (see Table 1). SDPC contains mixed chains (C22:6 (DHA) and C18:0), so these SDPC levels translate into a DHA content of 11% and 3% over total fatty acids, respectively (see Table 1). 33% cholesterol was also present in both membranes. Three replicas, each 60 μs long, were simulated for both compositions. Additionally, other similar systems were simulated to support the findings of this study. All these simulations, totalling to almost 4 ms, are summarized in Table S1 with their compositions given in Tables S2 and S3. We report all CG-MD simulation using effective times, a standard 4-fold speed-up conversion factor that accounts for the loss of friction in the MARTINI CG-MD model^36^.

### BRET experiments

HEK-293T cells were transiently transfected with a constant amount (0.3 μg) of 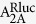 and increasing amounts of plasmid encoding 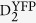, namely from 0.25 to 3.7 μg (see section 1.6 in the SI). The cDNAs encoding 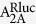, 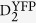 and CD4YFP were previously described in refs 39,40. Both fluorescence and luminescence signals from each sample were measured prior to experiments to confirm equal expression of the Rluc construct while monitoring the increase in YFP expression. Cells were then treated with DHA 200 μM for 48 h in the presence of adenosine deaminase (ADA, 0.2 U/mL) to remove any trace of adenosine from the culture medium, and were rapidly washed twice with phosphate-buffered saline, detached and re-suspended in the same buffer (see section 1.6 in the SI). This treatment resulted in the rise of DHA content from 0.99 ± 0.03% to 6.49 ± 0.32% (see section 1.6 in the SI). Triplicate samples of cell suspension (20 μg protein) were distributed in black bottom 96-well black microplates or white bottomed 96-well white microplates (Fisher Scientific, Madrid, Spain) for fluorescence or BRET experiments, respectively. For BRET measurements, colenterazine-h substrate (NanoLight Technology, Pinetop, Arizona, USA.) was added to a final concentration of 5 μM. BRET readings were performed at 1 and 10 min using the POLARstar Optima plate reader (BMG Labtech, Durham, NC, USA). This plate reader allows detection and sequential integration of both luminescence (Rluc) and fluorescence (YFP) signals by two filter settings: 440–500 nm and 510–560 nm windows to detect 485 nm (Rluc, donor) and 530 nm (YFP, acceptor) signals, respectively. The BRET ratio (i.e. the fluorescence signal over the luminescence signal) was defined as described previously^39^ and measured in 4 independent experiments where cells were treated with DHA. The values of BRET^max^ (i.e. the maximal signal reached at saturation) and BRET^41^ (i.e. BRET ratio giving 50% of the BRET^max^) were also calculated as in ref. 39. The statistical assessment of BRET^max^ and BRET^41^ values across experiments was performed using a paired *t* test comparing DHA-treated versus non-treated cells. An equivalent BRET protocol was employed for receptor homomerisation experiments, as described in ref. 28.

### All-atom simulations

The CHARMM36^42^, CHARMM36c^43^ and CHARMM27^44^ force fields were used to represent lipids, cholesterol and proteins, respectively. The adenosine A_2A_ receptor was embedded into an equilibrated healthy-like membrane patch (see Table 1 and Table S4). A production run of 4 μs in the was performed using the ACEMD simulation package^45^. A detailed description of the construction, simulation protocols, and analyses is provided in section 1 of the SI.

## Results

### *ω*-3 PUFA increases oligomerisation in CG-MD simulations

We quantified protein aggregation in healthy-like and diseased-like membranes by analysing the number of protein–protein contacts consolidated during the simulation (see Table S1 for number and length of simulations). First protein–protein contacts already occur within 5–10 μs and they seem rather unspecific. To make sure that mostly stable contacts are quantified, we analysed the last 20 μs of the 60 μs simulations. Analysis of all simulations shows that protein aggregation is significantly enhanced (i.e. ~20% higher) in healthy-like conditions (high DHA). Specifically, the mean number of protein–protein contacts per monomer is 1.48 ± 0.06 and 1.20 ± 0.08 in healthy-like (high DHA) and diseased-like (low DHA), respectively. This finding suggests that DHA plays an important role in the oligomerisation kinetics of A_2A_ and D_2_ receptors. It is worth noting though that due to the coarse level of description of the CG model and the restraints applied to preserve protein tertiary structure (see section 1.1 in the SI), our CG-MD simulations cannot capture conformational changes related to receptor activation.

### DHA treatment does not increase the amount of oligomers at equilibrium in living cells

**Figure 1:**
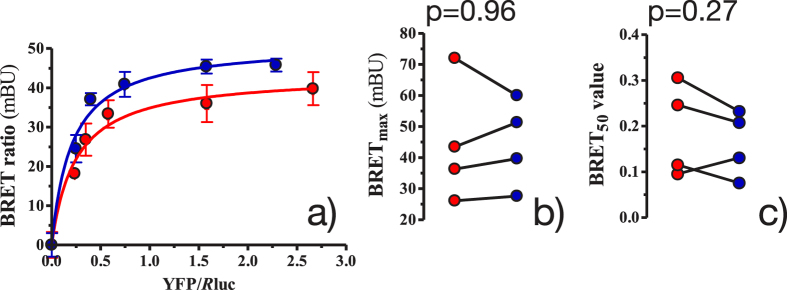
Effect of DHA on the amount of A_2A_–D_2_ heteromerisation in cellular steady state with low (red) and high (blue) DHA content. **(a)** shows representative BRET saturation curves, where each point measurement was performed in triplicate. BRET ratios (×1000) are in mBRET units (mBU). Error bars show the SEM. **(b**,**c)** columns show, respectively, the BRET^max^ and BRET^50^ results of 4 independent experiments with low (red) and high (blue) amounts of DHA. These results were compared by a paired *t* test and the *p* values are shown.

To study the effect of DHA on the steady-state kinetics of D_2_ and A_2A_ receptor oligomerisation, BRET experiments were carried out in living cells. In a first step, we demonstrated adequate incorporation of DHA into HEK-293T cell membranes (see section 1.6 in the SI). Then, we further investigated the role of membrane DHA in A_2A_ and D_2_ homo- and heteromerisation. BRET is a powerful technique for characterizing GPCR oligomers^46^ in the steady state albeit an accurate interpretation of the results is needed, as recently illustrated by Lan *et al.*^47^. In particular, BRET has been already useful for comparatively study the effect of certain modulators on GPCR oligomerisation^41^,^48^,^49^. In our experiments, a positive and saturable BRET signal for the transfer of energy between the acceptor 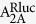 and the donor 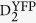 constructs was observed (Fig. 1a) in cells co-transfected with a constant amount of 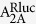 and increasing concentrations of 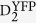. In addition, since the control receptor pair, 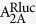 and CD4^YFP^, led to the typical quasi-linear curve^39^,^40^, the specificity of the saturation (hyperbolic) assay for the 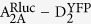 pair could be established. These results corroborate previous results indicating that A_2A_ and D_2_ receptors form constitutive heterodimers in living cells^39^.

To assess the effect of DHA on the A_2A_ − D_2_ heteromerisation (i.e. BRET signal), we performed 4 independent BRET titration experiments in the presence and absence of a saturating concentration of DHA (200 μM) (Fig. 1). Interestingly, as demonstrated by the large *p* values (i.e. *p* > 0.05) (Fig. 1b), preincubation with DHA for 48 h did not increase the maximum BRET signal (BRET^max^) in the experiments performed with 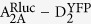 co-transfected cells. Similarly, DHA treatment did not have a systematic effect on the amount of acceptor-labelled receptor (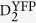) needed to reach 50% of the maximal BRET signal (BRET^50^) (see Fig. 1c). Therefore, DHA treatment was not found to affect the amount of heteromers present in steady-state conditions. Similarly, the number of A_2A_ − A_2A_ or D_2_ − D_2_ homodimers are not affected by membrane DHA, as shown in Fig. S23.

Overall, the computational results suggest that DHA treatment increases the kinetics of A_2A_ − D_2_ heteromerisation. Notably, additional simulations matching the exact content of DHA present in the experiments (see Table S1, Section 2.14 and Fig. S20) confirm an increased oligomerisation kinetics in the presence of higher levels of DHA. In contrast, the presence of this fatty acid does not regulate the amount of oligomers present in equilibrium, as shown by our BRET experiments.

### Polyunsaturated lipids avidly surround A_2A_ and D_2_ receptors in CG-MD and all-atom simulations

**Figure 2:**
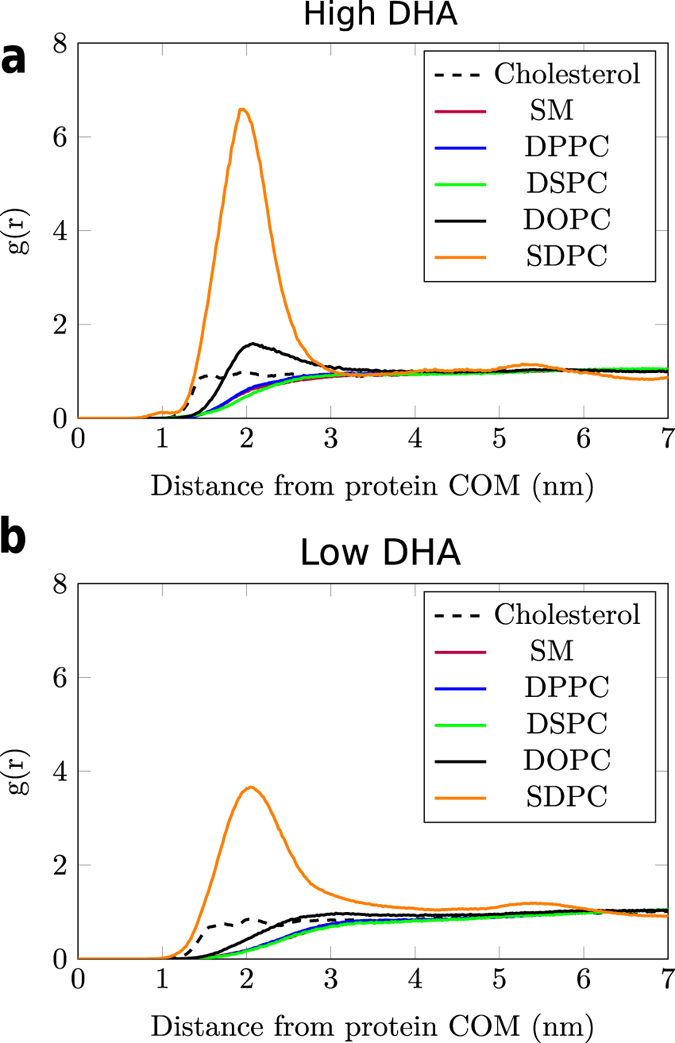
Probability density (i.e. radial distribution function, *g*(*r*)) of lipids around the center of mass (COM) of the A_2A_ receptor embedded in healthy- (high DHA, **(a)**) and diseased-like (low DHA, **(b)**) model membranes in CG-MD simulations. *y* axis represent *g*(*r*) (arbitrary units) and *x* axis the distance to the protein COM in nm. The radial distribution function of SM heavily overlaps with the rest of saturated lipids (i.e. DPPC and DSPC). Radial distribution functions for the D_2_ receptor are shown in Fig. S4.

To unlock the reasons that govern DHA-induced faster aggregation kinetics, MD simulations were employed to further characterize the affinity between DHA and A_2A_ and D_2_ protomers. As shown in the CG-MD simulations (see Movie S1), DHA-enriched phospholipids (i.e. SDPC) display a striking preference for interaction with A_2A_ and D_2_ receptors. In fact, this video clearly shows how a shell of this lipid surrounds GPCR monomers virtually from the beginning of the simulation, and how GPCR oligomers are still surrounded by SDPC molecules by the end of the simulation. To support these observations, we calculated the radial distribution function of each lipid type around the A_2A_ (see Fig. 2) and D_2_ (see Fig. S4) receptors. This analysis confirms that during CG-MD simulations the first solvation shell around the protein is primarily populated by phospholipids with DHA tails (SDPC). This DHA shell cannot completely form in the diseased-like systems (low DHA).

**Figure 3:**
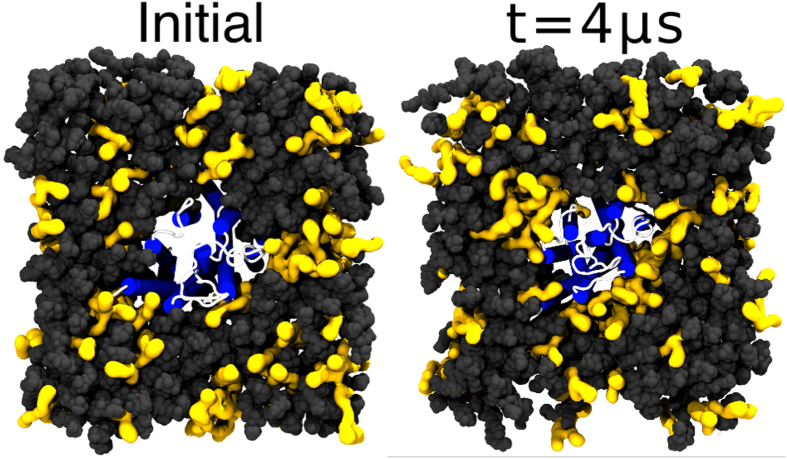
Initial and final (4 μs) snapshots of the all-atom simulation of the A_2A_ receptor embedded in a healthy-like membrane (high DHA). Protein helices are depicted in blue and loops in white. Unsaturated phospholipids (SDPC and POPC) are drawn as yellow surfaces with dark grey spheres corresponding to other lipid types. Water and ions are omitted for clarity.

**Figure 4:**
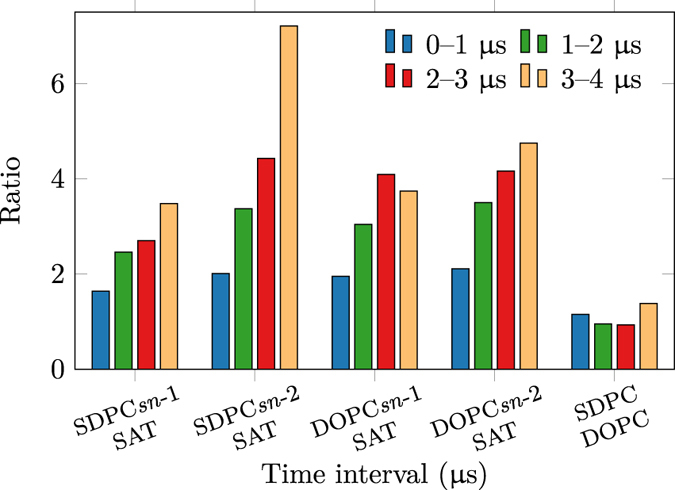
Ratios of A_2A_ receptor–lipid contacts of unsaturated (SDPC and POPC) and saturated (SAT) lipid tails during the all-atom simulation.

To validate this strong interaction between DHA and the receptors in the CG-MD simulations, we complemented these simulations using all-atom molecular dynamics of A_2A_ embedded in a healthy-like membrane system (see Table 1 and Table S4). A final snapshot of the all-atom simulation at 4 μs (Fig. 3, right) confirms that unsaturated phospholipids, namely DOPC and SDPC, have a strong preference for solvation of the protein. In agreement with previous simulations^19^, DHA does not exhibit affinity for a particular helix but rather solvates the A_2A_ receptor in a general fashion (see Fig. S6). Nevertheless, more atomistic simulations (i.e. more statistics) of both receptors are needed to draw consistent conclusions in this respect. To quantitatively assess this effect, we calculated the mean number of contacts per atom between unsaturated tails and the protein and compared this value with that for saturated tails (see Section 1.4.1 in the SI). The proportion of lipid–protein contacts between unsaturated tails with respect to saturated ones clearly grows during the simulation (see Fig. 4). Specifically, DHA tails (i.e. *sn*-2 chain of SDPC) display the highest growth rate (see Fig. 4) and confirm the tendency of this fatty acid to interact with the protein. In addition, the contact ratio of SDPC over DOPC remains equilibrated (i.e. around 1) until the end of the simulation, when, proportionally more SDPC is in contact with the protein (see Figs S5 and 4). These results imply that, as we observe in Fig. 3, DHA gradually populates the closest lipid shell around the A_2A_ during the all-atom simulation.

In addition, as shown in CG-MD simulations (Movie S1), an SDPC shell seems to act as a lubricating film in many of the dimer and oligomer formation events. It is tempting to suggest that the high affinity between DHA and GPCRs is responsible for the mechanism which fosters the oligomerisation kinetics of A_2A_ and D_2_ protomers. This effect might, however, not alter the number of oligomers present under equilibrium, as suggested by the BRET experiments.

### DHA accelerates the kinetics of GPCR oligomerisation

**Figure 5:**
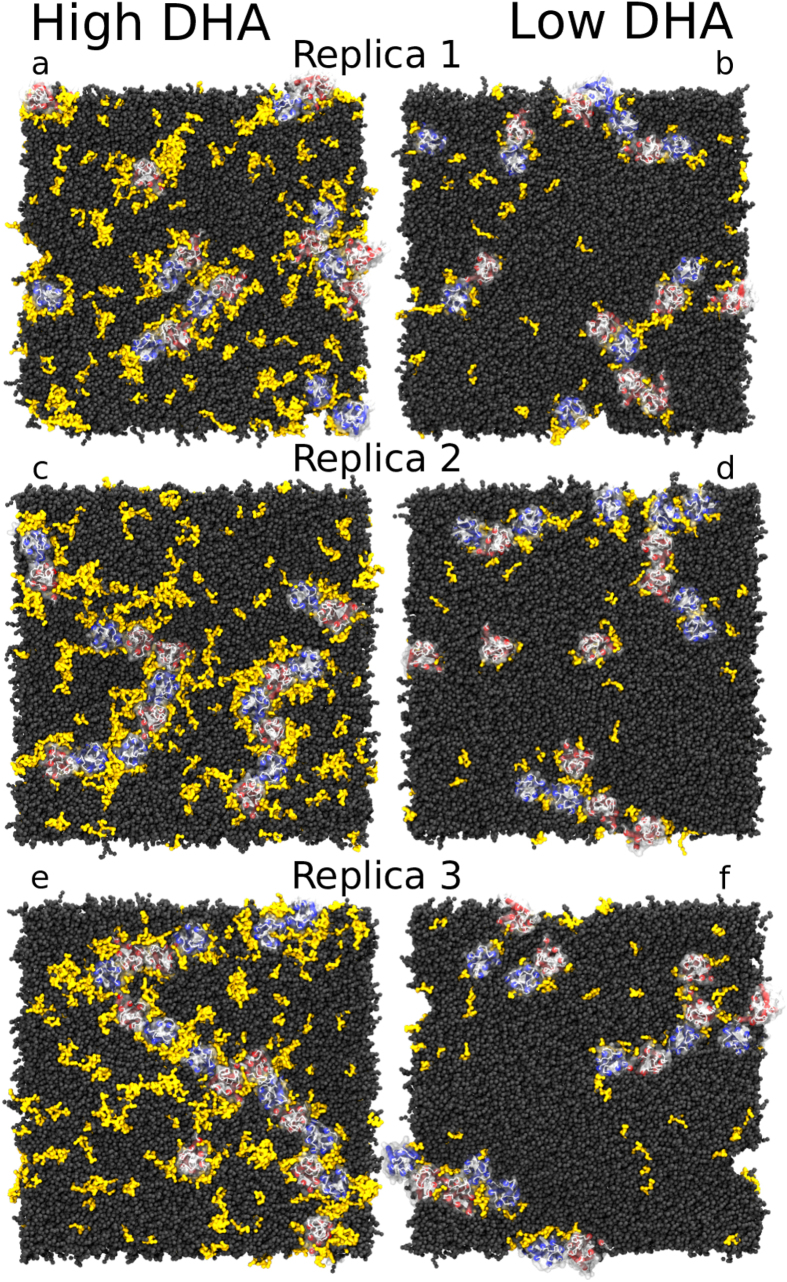
Final snapshots of healthy- and diseased-like systems after 60 μs of CG-MD simulation. Left and right columns display 3 replicas of healthy- (high DHA, left) and diseased-like (low DHA, right) systems. A_2A_ and D_2_ helices are depicted in red and blue cartoons, respectively. Dark grey spheres correspond to all membrane lipids except for SDPC molecules depicted in yellow surface. Water and ions are not shown for clarity.

We performed an in-depth characterization of the protein aggregation behaviour observed in CG-MD simulations. The final snapshots of these CG-MD simulations, shown in Fig. 5, indicate that protein oligomers tend to form extended rather than compact structures in both healthy-like and diseased-like environments. Within these oligomers, protomers establish mostly 1- (dimers) or 2-contacts (trimers) and only rarely 3-contacts (tetramers) with other protomers. Such array-like disposition has already been described in previous CG-MD simulations of GPCRs^50^,^51^. To confirm the behaviour of protein oligomers at longer timescales, we extended one of the simulations (i.e. initial snapshot shown in Fig. 5a) up to 260 μs. The final snapshot of this extended simulation, shown in Fig. S7d, displays an extended-like and short-branched arrangement of the protein oligomer where nearly all protomers form at least one protein–protein contact. It is worth noting that during these 60–260 μs, the number of protein–protein contacts generally remained between one and two contacts per protomer (see Fig. S7a–d and Section S2.5).

**Figure 6:**
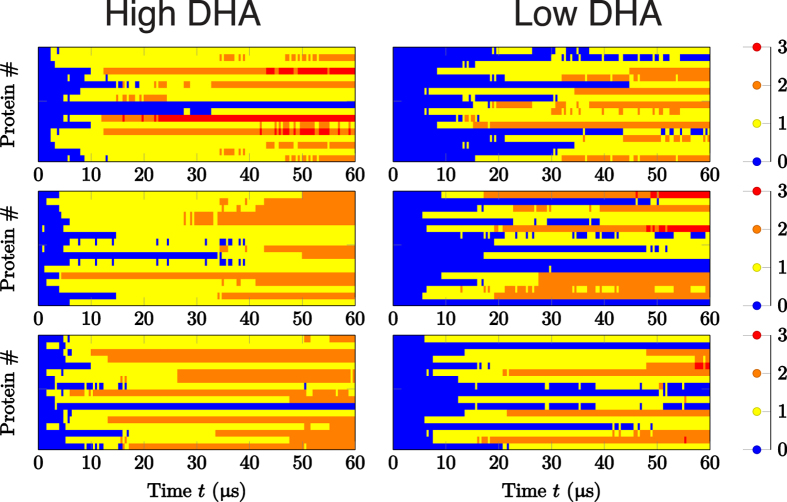
Time-dependence of protein aggregation in CG-MD simulations. Data are shown for healthy-like (i.e. high DHA, left) and diseased-like (low DHA, right) systems. Each cell represents one of the three replicas and each line in the plots corresponds to an individual receptor. The colour code reflects the number of contacts per protomer. Corresponding data for the short (16 μs) systems is shown in Fig. S9.

**Figure 7:**
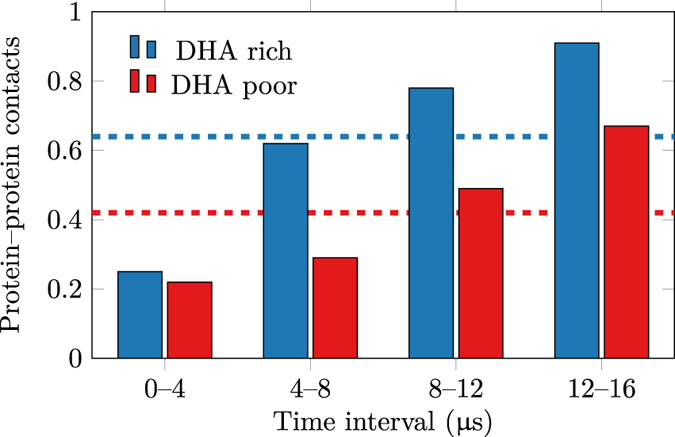
Time evolution of the mean number of protein–protein contacts per protomer in the set of shorter simulations. ‘Healthy’ and ‘Diseased’ refer to healthy-like (high DHA) and diseased-like (low DHA) model membranes. Average values are shown by dashed lines.

The evolution of protein aggregation significantly varied between healthy- and diseased-like systems, as shown by the number of protein–protein contacts over time depicted in Fig. 6. This figure shows that A_2A_ and D_2_ dimers form significantly quicker in high-DHA (Fig. 6, left column) systems when compared to low-DHA ones (Fig. 6, right column). To provide more statistical support for this finding, we performed 5 additional shorter replicas (see Table S1 for number and length of simulations) for both membrane compositions, obtaining a similar tendency (see Fig. S9). As shown in Fig. 7, the mean number of protein–protein contacts per protomer in these short replicas begin to deviate during the 4–8 μs interval of the simulation, as protomers embedded in healthy-like model membranes were able to engage in twice as many protein–protein contacts compared to diseased-like membranes. Therefore, increasing levels of membrane *ω*-3 PUFAs (i.e. DHA) seems to speed up GPCR oligomerisation by promoting a higher numbers of protein–protein contacts in shorter times. Two possible mechanisms might explain this tendency. First, the presence of DHA might enhance the rate of oligomerisation partner search through increased lateral diffusion. Second, oligomerisation might be fostered by the merging of the lubricating DHA shells of individual receptors. This process is possibly driven by the tendency of the membrane to separate into DHA-enriched and DHA-depleted domains. Likewise, the latter process competes with the propensity of DHA to interact with the receptors.

In the time scale of our simulations, as previously described in similar studies^50^,^52^,^53^, GPCR complexes do not tend to disrupt once they form. As a result, the initial arrangement of protomers likely determines the nature of the predominant interaction found at the end of the simulation (i.e. heteromers versus homomers). For example, at the end of the simulation (see Fig. 5), heteromers account for a major fraction of contacts in healthy and diseased-like systems. To further characterize this observation we performed new simulations using different initial arrangements of protein monomers (see section 2.6 and Table S1 in the SI for number and length of simulations). A protein–protein contact analysis on these simulations confirms that the enhancing effect of DHA on protein aggregation is not markedly driven by the initial configuration of protomers(see Tables S1 and S2 as well as Fig. S10). As shown in the SI (Figs S11 and S12), the DHA effect is also present when simulating receptors of the same protein type. Details about the preferred homo- and heterodimer interfaces formed can be found in the SI (section 2.2). In brief, as previously described in similar CG-MD studies^50^,^53^, oligomerisation of A_2A_ and dopamine D_2_ receptors predominantly occurs via TM1, TM2 and helix 8 or TM3, TM4 and TM5 surfaces.

### DHA increases lateral diffusion rates

To study the effect of DHA on protein diffusion, we performed a new set of CG-MD simulations based on single monomers (i.e. A_2A_ or D_2_) (see Table S1 for number and length of simulations) and extracted protein diffusion coefficients (see section 1.2.1 in the SI). As shown in Fig. S13, proteins display higher mean squared displacements (MSDs) when diffusing in the more fluid environment of a healthy-like (DHA-enriched) membrane than in diseased-like (low DHA) membranes. Specifically, A_2A_ and D_2_ receptors simulated in healthy-like membranes display an average diffusion coefficient of 4.8 ± 1.3 × 10^−9^ cm^2^/s and 4.6 ± 1.0 × 10^−9^ cm^2^/s, respectively. In contrast, when simulated in diseased-like membranes, A_2A_ and D_2_ receptors show a slower diffusion of 1.8 ± 0.6 × 10^−9^ cm^2^/s and 2.2 ± 0.6 × 10^−9^ cm^2^/s, respectively. Similarly, the calculated protein rotational motion is slower in diseased-like membranes (Fig. S14), a trend particularly evident for the D_2_ receptor. However, the effect of lipid composition on lipid diffusion are not markedly different across systems (Table S5). Therefore, while diseased-like (low DHA) environments slows down protein diffusion by a factor of 2–3 respect healthy-like (high DHA) one, differences in lipid diffusion coefficients are just within the error range.

In general, the simulation data suggest that higher levels of DHA allow proteins to travel longer distances and sample a higher number of potential dimerisation interfaces due to enhanced translational and rotational diffusion, respectively. As a result of a faster diffusion, proteins could aggregate more rapidly in healthy-like model membranes (Fig. 6, left), where we find that nearly all monomeric structures (blue) disappear within 5–10 μs, making room for dimers (yellow), trimers (orange) or even higher-order arrangements such as tetramers (red). In contrast, in diseased-like systems (Fig. 6, right), most of the monomeric structures need 15–20 μs to form higher-order structures.

To further study the dynamics of more crowded environments, we also calculated lipid diffusion coefficients in the multi-protein systems. Whereas lipid diffusion was not systematically affected by the presence of DHA in diluted conditions (non-crowded), lipids diffuse 10–40% faster in high-DHA membranes under protein-crowded conditions (see Table 2 and Fig. S15). In agreement with a recent study on lipid diffusion in protein-crowded environments^54^, lipid diffusion coefficients are, however, lower than those calculated from non-crowded simulations (see Table S5). It is worth noting that out of the six lipid species, SDPC displays the lowest diffusion coefficient in both healthy- and diseased-like systems (Table 2). Such low diffusion is consistent with the fact that SDPC is the most common lipid of the protein-solvating lipid shell (Figs 2 and S4). Moreover, practically all SDPC is bound to the receptors in diseased-like systems (i.e. low DHA) whereas a fraction of SDPC is freely diffusing in healthy like systems (i.e. high DHA) (Fig. 5). Hence, a plausible explanation for the low diffusion values shown by SDPC is that the high number of DHA–protein interactions slows down the diffusion of the bound SDPC, in agreement with previous work^55^. The comparison of lateral diffusion values obtained from these CG-MD simulations with experiments is discussed in section 2.17 of the SI.

### DHA–DHA interactions and lipid phase-segregation contribute to increase the oligomerisation rate

To better understand whether other factors besides increased lateral mixing contribute to the higher oligomerisation rate observed in healthy-like (i.e. high DHA) membranes, we performed further CG-MD simulations. We built an additional system using a simplified membrane composition even richer in DHA. Namely, we embedded 9 A_2A_ and 9 D_2_ protomers in a ternary mixture made of 1-palmitoyl-2-oleoyl-*sn*-glycero-3-phosphocholine (POPC): 1-stearoyl-2-docosahexaenoyl-*sn*-glycero-3-phosphatidylethanolamine (SDPE): cholesterol (30%:50%:20%) and simulated 3 replicas for 120 μs (see section 1.1.3 and Tables S1 and S3b in the SI). It is worth noting that in this system DHA accounts for up to ~31% of the lipid tails. Due to the lack of saturated lipids (DSPC, DPPC or SM) this system showed ~3 times faster diffusion values when compared to the diffusion results measured in conventional healthy-like systems. Thus, we obtained values of 8.1 ± 0.7 × 10^−8^ cm^2^/s, 8.1 ± 0.6 × 10^−8^ cm^2^/s and 12.4 ± 0.7 × 10^−8^ cm^2^/s for POPC, SDPE and cholesterol, respectively. However, faster lipid diffusion rates did not correlate with higher protein oligomerisation rates (see Fig. S16). Actually, in the membrane with very high DHA content most of the protein monomers take longer to engage in protein–protein interactions and many of them remain in a monomeric state after 120 μs. This result indicates that despite DHA concentration does modulate the mobility of membrane components, a higher mobility does not necessarily lead to an increased oligomerisation rate.

Moreover, to ascertain whether lipid-segregation forces contribute to the DHA effect, we studied the tendency of healthy-like membranes to undergo phase separation into DHA-enriched and DHA-depleted regions in the absence of proteins. Thus, we built a protein-free version of the healthy-like system (see Table S3a) and simulated it for 40 μs (see Table S1 for number and length of simulations). The ability of this lipid mixture to separate into domains could explain why DHA-coated receptors prefer to remain in close contact with each other. As shown in Fig. S17, healthy-like systems tend to separate into DHA-enriched and DHA-depleted domains. However, we do not observe any sharp domain boundary typically observed in phase separation studies using the Martini force field^56^. Instead, our simulations show a partial phase separation potentially induced by the hybrid nature of SDPC. We validated this observation by computing the time evolution of the contact fraction between saturated and unsaturated lipids as calculated in ref. 57 (see Fig. S18). Therefore, a partial phase-separation effect drives DHA-coated receptors to come closer to each other quicker than proteins where a DHA shell is not present or not entirely formed. Likewise, protein concentration will tend to be higher in DHA-enriched domains thus maximizing protein–protein interactions. Phase separation effects are absent in the system with very high DHA content.

In addition to limiting the accessible area of the receptors through partial phase separation, the favourable interaction between DHA chains likely contributes to the increased oligomerisation kinetics in high-DHA systems. DHA shells seem to increase the effective dimerisation radius of DHA-coated receptors when compared to uncoated ones. Thus, DHA–DHA interactions would enhance protein oligomerisation by allowing DHA-coated receptors to ‘sense’ each other at longer distances and eventually bringing receptors closer (see Movie S1 and section S2.1). We quantified this effect by assessing the receptor–receptor distance over time. Characteristic examples of these plots are shown in Fig. S19. In diseased-like membranes (i.e. low DHA), receptors can remain at an intermolecular distance of approximately 5 nm for several μs without effectively aggregating. In contrast, in healthy-like membranes (i.e. high DHA) effective oligomerisation consolidates as soon as protomers get closer than 5.5–6 nm. Interestingly, this value corresponds to the sum of the cut-off distance employed to define a protein–protein contact (4.2 nm) plus the widths of the two SDPC shells in the membrane plane (2 times approximately 0.8–0.9 nm).

Finally, we assured that the observed accelerated effect on receptor oligomerisation is not an artefact of overestimated protein–protein interactions of the Martini model^58^ and the use of Martini version 2.1 instead of the recently released version 2.2^59^. We verified that neither scaling down the protein–protein interactions nor using the latest version of the force fields affects this conclusion (see Section 2.15 and Table S1 in the SI for number and length of simulations).

Overall, these results indicate that favourable DHA–DHA interactions and lipid phase-segregation forces contribute along with increased lateral mixing to the effect of DHA on protein aggregation.

## Discussion

Experimental evidence suggests that both GPCR oligomerisation^22^,^23^,^24^,^25^ and DHA^4^,^5^,^6^ play a relevant role in brain functioning. In this study we report for the first time a molecular link between membrane levels of DHA and the oligomerisation rate of A_2A_ and D_2_ receptors, which could have important implications for the treatment of major psychiatric disorders.

We have used molecular simulation methods and laboratory experiments to assess the effect of DHA levels on the formation of A_2A_ − D_2_ oligomers. CG-MD simulations indicate that the kinetics of adenosine A_2A_ and dopamine D_2_ aggregation is modulated by membrane DHA levels. Specifically, low levels of DHA significantly diminished the ability of A_2A_ and D_2_ receptors to engage in protein–protein contacts in the microsecond time scale probed by these simulations. To further study the impact of low and high DHA levels on GPCR oligomerisation, we used a classical experimental approach, namely BRET measurements in living cells. It is important to note that the temporal sensitivity of the BRET technique is around the millisecond time scale, thus reaching far beyond our CG-MD simulations (i.e. microseconds). Accordingly, we employed BRET determinations to monitor DHA-mediated changes upon equilibrium conditions through end-point experiments. These experiments show that the level of membrane DHA does not affect the steady-state intensity of A_2A_ − D_2_ oligomerisation (Fig. 1), thus suggesting a purely kinetic effect of membrane DHA on receptor oligomerisation. While DHA has an important role in the speed of oligomer formation in CG-MD simulations, higher levels of this fatty acid does not seem to impact the amount of oligomers in equilibrium. Nevertheless, caution is needed when interpreting results as the BRET approach might not be sufficiently sensitive to pick up a DHA-induced alteration on the equilibrium state of receptor oligomerisation. New imaging-based approaches with higher temporal resolution (e.g. double-receptor tracking by super resolution fluorescence microscopy) should, however, be implemented in the near future to solve the sensitivity issue revolving around the BRET approach.

In addition, this study provides insights into a mechanism that could underlie the kinetic effect of DHA on receptor oligomerisation. We find that membrane DHA levels modulate the diffusion of membrane lipids (Table 2, Table S5 and Fig. S15 in the SI), the diffusion of receptors (Table S5 and Figs S13 and S14) and, ultimately, the rate of spontaneous protein–protein interactions (Fig. 6). Thus, A_2A_ and D_2_ receptors can travel longer distances and sample a higher number of potential dimerisation interfaces in high-DHA membranes. Unexpectedly, control simulations using a very high DHA content show drastically higher values of lipid diffusion which does not correlate with increased oligomerisation rates (Fig. S16). This suggests that diffusion rates are not the only contributing factor to DHA-induced effect on protein oligomerisation. Interestingly, the formation of a DHA shell around the receptors seems to play a key role in this effect (see Movie S1). The presence of this solvation shell (see Figs 2 and S4 as well as Fig. 4) in both CG-MD and all-atom MD simulations is in line with previous experiments^60^ and simulations^19^ showing a preferential aggregation of DHA around rhodopsin. The high affinity between DHA and receptors is based on the tremendous flexibility of DHA tails when compared to saturated and mono-unsaturated ones^61^. As demonstrated by Grossfield *et al.*^20^, the nature of the spontaneous solvation of GPCRs by DHA relies on the lower entropic cost paid by polyunsaturated tails like DHA to interact with the protein.

We propose that this shell is involved in two fundamental properties of the enhancing effect of DHA on protein oligomerisation. Firstly, control simulations demonstrate that DHA can induce partial phase segregation (Figs S17 and S18), in agreement with previous experiments^9^. The low affinity between DHA and the rest of membrane lipids including cholesterol is a major entropic factor leading to phase segregation and ultimately to the enrichment of DHA around receptors, as previously shown for rhodopsin^62^. In this scenario, receptors covered by DHA will partition into DHA-enriched domains. This reduces the effective sampling area of receptors within the membrane thus increasing the number of receptor–receptor encounters. Secondly, DHA shells enhance the ability of DHA-coated receptors to engage in protein–protein contacts by increasing its effective oligomerisation radius. As a result, protein oligomerisation does not necessarily need direct protein–protein contacts to occur but it is rather initiated as two DHA shells come closer due to the high affinity between DHA tails (see Movie S1 and Fig. S18). Both effects (i.e. phase segregation and favoured interactions between DHA-coated receptors) are negligible when DHA is present in too high concentrations. In this scenario, no membrane domains are formed and DHA-coated receptors are not particularly driven to engage in protein–protein contacts as they equally interact with the DHA-enriched membrane bulk. Overall, these results suggest that the role of DHA on receptor aggregation kinetics is beyond increasing membrane fluidity but rather based on both an increased lateral mobility of receptors and a favoured interaction between proteins fostered by the presence of a DHA solvation shell. Ultimately, a relevant question arises about how this mechanism translates into a specific receptor signalling response. As postulated by Noé and co-workers^63^, the formation of specific supramolecular architectures (i.e. tracks of rhodopsin dimers) does not seem to accelerate the rate of G protein binding to rhodopsin. However, these oligomers are thought to create a kinetic trap for G_t_ molecules controlling the rate of rhodopsin activation thus modulating receptor signalling^64^. In this context, it tempting to speculate that a lack of DHA (i.e. as observed in certain neuropsychiatric disorders) could impair the efficiency of this kinetic trap by diminishing the formation rate of specific signalling platforms such as A_2A_ and dopamine D_2_ receptor oligomers.

In conclusion, our results suggest that higher levels of DHA accelerate protein aggregation and highlights the role of kinetics in modulating A_2A_ and D_2_ oligomerisation. Through extensive simulations on a wide set of model systems, we have been able to postulate a mechanism behind the influence of membrane DHA on the oligomerisation kinetics of A_2A_ and D_2_ receptors. These results provide an important advance in understanding the interplay between membrane lipids and key transmembrane proteins like GPCRs, a topic of current special interest in biochemistry and biophysics. Importantly, these results could partly underlie the neuroprotective properties of DHA supplementation reported previously in animal studies^5^,^12^. In particular, the observed kinetic effect could have an impact in the response rate to endogenous neurotransmitters in patients with an altered DHA membrane content. In that case, restoring membrane DHA levels in individuals with schizophrenia or Parkinson’s disease could become a strategy to improve the impaired crosstalk of the A_2A_ − D_2_ oligomer observed in these disorders^23^. Our findings create new opportunities to explore the use of membrane lipids as a therapeutic tool for major neuropsychiatric conditions where A_2A_ and D_2_ oligomers have been shown to have particular importance.

## Additional Information

**How to cite this article**: Guixà-González, R. *et al.* Membrane omega-3 fatty acids modulate the oligomerisation kinetics of adenosine A_2A_ and dopamine D_2_ receptors. *Sci. Rep.* **6**, 19839; doi: 10.1038/srep19839 (2016).

## Supplementary Material

Supplementary Information

Supplementary Movie S1

## Figures and Tables

**Table 1:** Phospholipid and fatty acid compositions of healthy- and diseased-like model membranes used in all simulations.

Phospholipid (tails)	Healthy (%)	Diseased (%)
DPPC (diC16:0)	21	33
DSPC (diC18:0)	7	15
DOPC (diC18:1)	15	11
SDPC (C22:6/C18:0)	21	6
SM (C18:1/C16:0)	36	36
**Fatty acid**	**Healthy (%)**	**Diseased (%)**
C16:0	39	51
C18:0	43	41
C18:1	7	5
C22:6	11	3

Both membranes also contained 33% cholesterol. Abbreviations signify 1,2-dipalmitoyl-*sn*-glycero-3-phosphocholine (DPPC), 1,2-distearoyl-*sn*-glycero-3-phosphocholine (DSPC), 1,2-dioleyl-*sn*-glycero-3-phosphocholine (DOPC), 1-stearoyl-2-docosahexaenoyl-*sn*-glycero-3-phosphocholine (SDPC) and sphingomyelin (SM).

**Table 2:** Diffusion coefficients of lipids extracted from the multi-protein CG-MD systems.

Lipid	Healthy	Diseased
CHOL	3.4 ± 0.2	2.6 ± 0.2
SM	2.7 ± 0.2	2.5 ± 0.2
DPPC	2.9 ± 0.4	2.5 ± 0.2
DSPC	2.9 ± 0.2	2.4 ± 0.1
DOPC	2.9 ± 0.3	2.4 ± 0.1
SDPC	2.1 ± 0.2	1.3 ± 0.1

‘Healthy’ and ‘Diseased’ refer to healthy-like (high DHA) and diseased-like (low DHA) model membranes. Values are reported in 10^−8^ cm^2^/s ± the error estimate. The calculation of error estimates is described in section 1.2.1 of the SI.
