# Supplementary material for: Membrane omega-3 fatty acids modulate the oligomerisation kinetics of adenosine A2A and dopamine D2 receptors
Source: Sci Rep. 2016 Jan 22;6:19839. doi: 10.1038/srep19839 (PMC4726318; doi:10.1038/srep19839)
Supplement: Supplementary Information [file srep19839-s1.pdf]

Electronic Supplementary Information
Membrane omega-3 fatty acids modulate the oligomerisation kinetics of adenosine A_{2A} and dopamine D_2 receptors

Ramon Guixà-González[a,‡], Matti Javanainen[b,‡],
Maricel Gómez-Soler[c], Begoña Cordobilla[d],
Joan Carles Domingo[d], Ferran Sanz[a],
Manuel Pastor[a], Francisco Ciruela[c,e],
Hector Martinez-Seara[b,*], Jana Selent[a,*]

November 14, 2015

[a] Research Programme on Biomedical Informatics (GRIB),
Department of Experimental and Health Sciences Universitat Pompeu Fabra,
IMIM (Hospital del Mar Medical Research Institute), Spain
[b] Department of Physics, Tampere University of Technology, Tampere, Finland
[c] Facultat de Medicina, IDIBELL, Universitat de Barcelona, Barcelona, Spain
[d] Facultat de Biologia, Universitat de Barcelona, Barcelona, Spain
[e] Faculty of Sciences, University of Ghent, Gent, Belgium
[‡] These authors contributed equally to this work
[*]hseara@gmail.com, jana.selent@upf.edu

Abstract

This Supporting Information (SI) document provides detailed information on the construction of the coarse-grained and all-atom simulations employed to study receptor oligomerisation. Additionally, the analysis performed on the data obtained from these simulations is described. This SI also contains a set of additional results which complement those presented in the main text.

1 Methods

1.1 Coarse-grained (CG) molecular dynamics simulations

1.1.1 Coarse-grained models of A_{2A} and D_2 receptors

While a crystal structure[1] (PDB:3EML) was directly used as an all-atom representation of the adenosine A_{2A} receptor, a homology model of the D_2 receptor was built based on the crystal structure of the highly homologous dopamine D_3 receptor (PDB:3PBL). Missing residues were modelled using MODELLER[2], and the intracellular loop 3, not resolved in any of the current GPCR crystal structures, was omitted in both cases. All titratable residues were left in the dominant protonation state at pH 7.0. A structure and the corresponding topology compatible with Martini force field v2.1[3] were created based on the former atomistic structure files using the `martinize` tool. To preserve the tertiary structure of proteins during the simulation, an elastic network was applied between beads as a structural scaffold following the ElNeDyn approach[4]. Based on the fluctuations observed in C_α atoms during all-atom simulations of A_{2A} and D_2 receptors, force constants of 1000 kJ/mol and 250 kJ/mol were applied to the helical regions and the loops, respectively.

1.1.2 Construction of the CG systems

Inspired by brain postmortem studies of healthy and diseased subjects[5–10], we built two multi-component lipid bilayers, namely 'healthy-like' and 'diseased-like' model membranes. To keep an adequate balance between lipid components we followed key general tendencies observed in the former studies rather than exact proportions. The aim was to create two native-like model membranes, one DHA-high (healthy-like) and one DHA-low (diseased-like), by modifying DHA levels while preserving adequate amounts of other relevant membrane components (i.e. saturated lipids, monounsaturated lipids and cholesterol). In short, two lipid bilayers were created by arranging 450 lipids[11] randomly to form a symmetric bilayer. The relative lipid composition of each bilayer is given in Table 1 the main text (see also Table S2a). These bilayers were subsequently solvated and 10% of the water was replaced by the antifreeze water of the Martini force field. This was followed by an equilibration phase. Thereafter, both two receptors were simultaneously embedded into the equilibrated lipid bilayers without compromising the lipid composition[12]. Protein excess charge was neutralized with chloride beads. Subsequently, larger systems were created based on these patches by replicating their contents independently 9 times (3 × 3) in the membrane plane using the `genconf` utility of GROMACS[13]. The composition of these constructed systems is detailed in Table S2a.

1.1.3 Simulations of CG systems

The systems were first equilibrated with protein beads constrained. Eight different starting structures were then constructed to be simulated as independent replicas for both compositions (i.e. healthy- and diseased-like). The initial coordinates for these replicas were extracted at large time intervals of 2 to 8 μs from the simulation where protein beads were constrained. This enabled a proper mixing to the lipid environment surrounding each protein. For each membrane type, three replicas were simulated for 60 μs ('Long initial' in Table S1) while the remaining 5 replicas were simulated for 16 μs ('Short initial' in Table S1) to validate the effect of DHA at shorter times. One healthy-like simulation was extended up to 260 μs to observe the arrangement of protein oligomers at longer time-scales ('Extended Initial' in Table S1). Likewise, a similar pair of the initial systems (i.e. 3 × healthy- and 3 × diseased-like)

were subsequently built following the same protocol but using a different initial arrangement of protein monomers ('Validation' in Table S1). Furthermore, to study the effect of membrane composition on the homomerisation of A_{2A} and D_2 receptors, two new systems containing either A_{2A} or D_2 receptors were built and three replicas of each of the systems (i.e. 3 × healthy- and 3 × diseased-like) were simulated for 120 μs ('Homomers' in Table S1). The DHA content employed in BRET experiments was somewhat smaller than in the simulations of 'Initial' and 'Validation' systems. Therefore to enable proper comparison between experiments and simulation, additional 5 short replica simulations with the exact DHA concentration employed in the experiments were performed. The compositions of these systems were generated by modifying the amounts of SDPC and DSPC starting from the healthy-like and diseased-like compositions used in the initial simulations. These systems are referred to as 'BRET composition' in Table S1 in the manuscript. The exact composition of all these simulations is detailed in Table S2.

Additionally, single protein diffusion was studied by simulating monomeric A_{2A} and D_2 receptors independently embedded in the initially constructed small membrane patches of both healthy- and diseased-like compositions (i.e. the 1/9th of the lipids given in Table S2a). These systems were simulated for 32 μs and referred to as 'Single-protein' in Table S1. Additionally, a protein-free bilayer ('Protein-free' in Table S1) with the healthy-like lipid composition given in Table S3a was simulated for 40 μs in order to study its phase behaviour. Finally, three replicas of a membrane with high DHA content, whose composition is given in Table. S3b, were simulated for 120 μs. 'Very high DHA' in Table S1 refers to this system. All the simulations listed above are summarized in Table S1. All files required to reproduce all simulations have been deposited in the Zenodo repository (`http://dx.doi.org/10.5281/zenodo.33491`).

Table S1: Summary of the performed CG-MD simulations. The bold number 2 refers to the two compositions (healthy-like and diseased-like), whereas numbers in normal font refer to the replicas. In the 'Single-protein' systems, however, 2* refers to the A_{2A} and D_2 receptors being simulated separately. The total simulation time is almost 4 ms

**Title**	**Composition**	**Simulations**	**Purpose**
Long initial	Tab. S1a	$\mathbf{2} \times 3 \times 60$ μs	Calculate amount of oligomers
Short initial	Tab. S1a	$\mathbf{2} \times 5 \times 16$ μs	Short-time oligomerisation kinetics
Extended initial	Tab. S1a, **Healthy**	1×260 μs	Long time oligomer rearrangement
Validation	Tab. S1b	$\mathbf{2} \times 3 \times 120$ μs	Test different initial arrangement
Homomers	Tab. S1c	$2^* \times \mathbf{2} \times 3 \times 120$ μs	Calculate amount of homomers
BRET composition	Tab. S1d	$\mathbf{2} \times 5 \times 20$ μs	Match experimental DHA content
Protein-free	Tab. S2a	1×40 μs	Study membrane phase behaviour
Very high DHA	Tab. S2b	3×120 μs	Further study DHA effect
Single-protein	Tab. S1a (1/9th)	$2^* \times \mathbf{2} \times 1 \times 32$ μs	Study diffusion of receptors

1.1.4 CG simulation protocol

All simulations were performed using the GROMACS 4.5.X simulation package[13] using a time step of 10 fs in the NPT ensemble. The temperature was kept constant at 310 K with the Berendsen thermostat[14] using a relaxation time constant of 1 ps. Membrane and solvent components were coupled separately. The pressure was coupled semi-isotropically with the Berendsen barostat[14] using a relaxation time constant of 5 ps and a reference pressure of 1 bar. The shift approach was employed for non-bonded interactions. The electrostatic interactions were shifted to zero between 0 and 1.2 nm whereas for Lennard-Jones interactions the shifting was conducted between 0.9 and 1.2 nm. The neighbour list with a radius of 1.2 nm was updated every 10 steps. Periodic boundary conditions were employed in all three dimensions.

Table S2: Number of residues comprising all sets of CG simulations. Composition of **(a)** initial simulations, **(b)** validation systems, **(c)** the simulations of homomerisation, and **(d)** the simulations mimicking the amount of DHA present in the experiments. Lipid compositions yield protein to phospholipid ratios of 1:151 in **(a)**, 1:152 in **(b)**, 1:170 in **(c)** and 1:151 in **(d)**. Note that 'Homomers' simulations include 4 systems: A_{2A} in healthy and diseased lipid compositions and the same for D_2. Abbreviations stand for 1,2-dipalmitoyl-*sn*-glycero-3-phosphocholine (DPPC), 1,2-distearoyl-*sn*-glycero-3-phosphocholine (DSPC), 1,2-dioleyl-*sn*-glycero-3-phosphocholine (DOPC), 1-stearoyl-2-docosahexaenoyl-*sn*-glycero-3-phosphocholine (SDPC), 1-palmitoyl-2-oleoyl-*sn*-glycero-3-phosphocholine (POPC), 1-stearoyl-2-docosahexaenoyl-*sn*-glycero-3-phosphatidylethanolamine (SDPE), sphingomyelin (SM), cholesterol (CHO), water beads (W) and Martini antifreeze particles (WF), respectively.

	**(a):** Initial		**(b):** Validation	
	**Healthy**	**Diseased**	**Healthy**	**Diseased**
DPPC	576 (14%)	900 (22%)	512 (14%)	832 (22%)
DSPC	198 (5%)	396 (10%)	192 (5%)	384 (10%)
DOPC	396 (10%)	288 (7%)	352 (10%)	288 (7%)
SDPC	576 (14%)	162 (4%)	512 (14%)	128 (3%)
SM	972 (24%)	972 (24%)	864 (24%)	960 (25%)
CHO	1332 (33%)	1332 (33%)	1184 (33%)	1280 (33%)
Total	4050	4050	3616	3872
A_{2A}	9	9	8	8
D_2	9	9	8	8
W	44865	44028	44488	48360
WF	5004	4905	4960	5384
Cl^-	153	153	136	136

	**(c):** Homomers		**(d):** BRET composition	
	**Healthy**	**Diseased**	**Healthy**	**Diseased**
DPPC	576 (14%)	900 (22%)	576 (14%)	900 (22%)
DSPC	198 (5%)	396 (10%)	421 (10%)	504 (12%)
DOPC	396 (10%)	288 (7%)	396 (10%)	288 (7%)
SDPC	576 (14%)	162 (4%)	353 (9%)	54 (1%)
SM	972 (24%)	972 (24%)	972 (24%)	972 (24%)
CHO	1332 (33%)	1332 (33%)	1332 (33%)	1332 (33%)
Total	4050	4050	4050	4050
A_{2A}	16 or 0	16 or 0	9	9
D_2	0 or 16	0 or 16	9	9
W	44560	48240	44865	44028
WF	4976	5376	5004	4905
Cl^-	160	160	153	153

Table S3: The composition of 'Protein-free' and 'Very high DHA' control CG simulations are given in **(a)** and **(b)**, respectively. In brackets, lipid percentages over total number of lipids.

(a): 'Protein-free' simulation

DPPC	576 (14%)
DSPC	198 (5%)
DOPC	396 (10%)
SDPC	576 (14%)
SM	972 (24%)
CHO	1332 (33%)
Total	4050 (100%)
A_{2A}	0
D_2	0
W	44865
WF	5004
Cl^-	153

(b): 'Very high DHA' simulations

POPC	1224 (30%)
SDPE	2016 (50%)
CHO	810 (20%)
Total	4050 (100%)
A_{2A}	9
D_2	9
W	80964
WF	9009
Cl^-	153

1.2 Analysis of the CG-MD simulations

1.2.1 Protein and lipid diffusion

The g_msd tool of GROMACS package[13] was used to perform all mean squared displacement (MSD) calculations. Lipid lateral diffusion was studied in multi-protein systems once most of the protein–protein contacts were established. Thus, the last 30 μs of the trajectories were employed for the analysis. All lipid species were considered separately and the possible drift of the system was eliminated by considering the motion of lipids with respect to the center of mass of the membrane. Lipid diffusion coefficients were extracted as the slope of a linear fit to the calculated MSD data, as defined in Eq. (1):

$$D = \lim_{\tau \to \infty} \frac{\mathrm{MSD}(\tau)}{4\tau} = \lim_{\tau \to \infty} \frac{\langle [\vec{r}(t+\tau) - \vec{r}(t+0)]^2 \rangle_t}{4\tau}, \tag{1}$$

where t is the simulation time and τ is the lag time. The angle brackets denote averaging over both time t and the studied molecules. Practically, instead of a limiting value at infinite time, the diffusion coefficients were extracted from linear fits, which were performed to the MSD curve in the lag time interval between 3 and 27 μs. This fitting interval was also used to calculate the diffusion coefficients of the 'Very high DHA' system.

Protein lateral and rotational diffusion was studied in the single-protein systems described earlier. Protein lateral diffusion coefficients were obtained from a linear fit of equation (1) to the lag time interval of 0.8–4 μs. In the calculation of both protein and lipid diffusion coefficients, error estimates were reported as the difference of the diffusion coefficients obtained from fits to two halves of the whole fit interval. Additionally, lipid diffusion coefficients were extracted also from these single-protein systems. In this case, the fits were performed to lag time interval between 0.8–16 μs.

Rotational diffusion of proteins was also studied on single-protein simulations using the g_rotacf tool of GROMACS[13]. The 2nd order Legendre polynomial was employed.

1.2.2 Radial Distribution Functions (RDFs)

The RDFs of lipids around proteins were calculated using the `g_rdf` tool of GROMACS[13]. RDF values are reported as averages over the three 60 µs replicas for both compositions.

1.3 All-atom molecular dynamics simulations

1.3.1 Construction of all-atom systems

A healthy-like model membrane (see Table 1 in the manuscript) of approximately 100 × 100 $Å^2$ (in the membrane plane) was built using the CHARMM-GUI membrane builder[15]. Since sphingomyelin was only introduced in CHARMM in mid-2014[16], a VMD 1.9[17] script was employed to mutate the pertinent fraction of DSPC molecules into 18-carbon sphingomyelin (SM). SM topology and additional parameters were created by a generalization approach using the CGenFF force field[18,19] version 2b6 using the CHARMM ParamChem interface v0.9.1 (`https://www.paramchem.org`). No high penalty scores were obtained in the former process. Thereafter, a VMD 1.9[17] script was used to re-hydrate the membrane patch using approximately 30 water molecules (TIP3P model) per lipid. A concentration of 150 mM of NaCl was added into the system. With the recent introduction of SSM in CHARMM-GUI we have validated our initial setup by simulating the same system again using the official SSM parameters. However, no changes where observed (see details below).

Table S4: Number of residues comprising the atomistic simulation. The number of residues of each component is shown. In brackets, lipid percentages over total number of lipids. The lipid composition yields a protein–to–lipid ratio of 1:337. Abbreviations stand for for 1,2-dipalmitoyl-*sn*-glycero-3-phosphocholine (DPPC), 1,2-distearoyl-*sn*-glycero-3-phosphocholine (DSPC), 1,2-dioleyl-*sn*-glycero-3-phosphocholine (DOPC), 1-stearoyl-2-docosahexaenoyl-*sn*-glycero-3-phosphocholine (SDPC) and sphingomyelin (SM), cholesterol (CHO) and water (W), respectively.

**Component**	**Number**
DPPC	48 (15 %)
DSPC	18 (6 %)
DOPC	36 (11 %)
SDPC	44 (14 %)
SM	79 (24 %)
CHO	112 (34 %)
Total	337 (100 %)
A_{2A}	1
W	22908
Na^-	65
Cl^-	75

1.3.2 All-atom simulation protocol

First, the membrane was equilibrated for 1 μs in the NPT ensemble. Next, the crystal structure of the adenosine A_{2A} receptor[1] (PDB:3EML) was manually embedded into the equilibrated membrane patch using VMD1.9[17]. This protein structure was prepared as described earlier. The final combined membrane–protein system comprised a total of 112,357 atoms. Table S4 gives a detailed composition of such system.

An NPT equilibration phase was then carried out so that lipids and water molecules could accommodate to the protein. To this end, harmonic positional restraints were applied to the C_α atoms of the protein and the system was simulated for 10 ns. Such constraints were gradually released from the receptor over 5 ns and the system was further equilibrated for 100 ns. In the production run, we simulated the system for 4 μs in the NVT ensemble. The ACEMD simulation package[20] was used. NPT simulations were carried out at 310 K and 1 bar using the Berendsen barostat[14] with a relaxation time of 400 fs and 2 fs integration time step. NVT simulations were run at 310 K, using the Langevin thermostat[21] with a damping coefficient of 5 ps^{-1} and 4 fs integration time step. In all simulation phases, van der Waals and short-range electrostatic interactions were cut off at 9 Å and the particle mesh Ewald method[22] was used to compute the long-range electrostatic interactions. A second 2 μs replica of the system was simulated to validate our all-atom protocol. In this replica, we used the SSM sphingolipid parameters provided by CHARMM-GUI which became available after a recent update. This system was simulated with GROMACS 5.0.4 in the NPT ensemble. We used all simulation parameters recommended and provided by CHARMM-GUI for simulation using GROMACS. No major differences were observed upon comparison of both systems, besides a slight reduction on the lateral area per lipid (data not shown). This effect has already been reported for other membrane systems and seems related to the implementation details of the CHARMM force field in GROMACS[23].

1.4 Analysis of the all-atom simulations

1.4.1 Lipid–protein contact ratios in all-atom MD simulations

We measured the ratio of lipid–protein contacts between two groups (e.g. two lipid species) by calculating the number of contacts per atom of the first group divided by the number of contact per atom of the second group. Atoms were considered in contact when located closer than 4.2 Å of the centre of mass of the protein. For this analysis only atoms in the lipid tails are considered. To facilitate the interpretation of the results, each contact value was previously normalized by the total number of atoms belonging to that particular selection. For example, in the SDPC/SAT ratio, SDPC is calculated as the number of atoms of any SDPC chain $<$ 4.2 Å of protein's centre of mass divided by the total number of SDPC chain atoms in the system. Likewise, SAT is calculated in the former ratio as the number of atoms of any saturated chain closer than 4.2 Å of protein's centre of mass divided by the total number atoms of saturated chains in the system.

1.4.2 3D density maps of DHA tails

The volumetric maps of DHA tails averaged during the 4 μs trajectory were computed using the Volmap Tool (`http://www.ks.uiuc.edu/Research/vmd/plugins/volmapgui/`) of VMD 1.9[17]. Default parameters were employed. All frames were computed and combined to report the average.

1.5 Figure art

The built-in Tachyon ray tracer of VMD 1.9[17] was used to render all snapshots from both CG and all-atom simulations. The pgfplots LaTeX package and the ggplot2 R package[24] were used to generate all plots.

1.6 Experimental protocol

1.6.1 Cell culture and transfection

Human embryonic kidney 293T (HEK-293T) cells were grown at 310 K in an atmosphere of 5 % CO_2 in Dulbecco's modified Eagle's medium (Sigma-Aldrich, St. Louis, MO, U.S.A.) supplemented with 1 mM sodium pyruvate, 2 mM L-glutamine, 100 mg/mL streptomycin, 100 U/mL penicillin and 5 % (v/v) fetal bovine serum. The cells were seeded into six-well plates at 300,000 cells/well and transiently transfected with the corresponding cDNA constructs using Transfectin (Bio-Rad, Hercules, CA, U.S.A.) following manufacturer's instructions.

1.6.2 DHA fatty acid supplementation and cell viability assay

Triglyceride fish oil was kindly provided by Brudy Technology (Barcelona, Spain). This oil contains more than 70 % of DHA in total fatty acids and more than 90 % ω-3 fatty acid triglycerides. Transfected cells were incubated with media containing different amounts of DHA (namely 5, 10, 20, 30, 50, 100 and 200 µM) for 48 h. To determine the effect of DHA on cell viability we used the CALcein-AcetoxyMethyl Ester Diacetate (CAL-AM), a cell-permeable dye (EMD Millipore, MA, USA). In brief, HEK-293T cells (1×10^4 cells/well) were cultured in a 96-well plate at 310 K, and exposed to varying concentrations of DHA for 48 h. Cells treated with plain medium served as a negative control group, whereas cells warmed at 338 K for 1 min were used as a positive control of cell death. After removing the supernatant of each well and washing twice with PBS, a solution of 100 µl of 1.0 µM calcein-AM diluted in warm (310 K) PBS was added to cells. After an incubation period of 30 min at 310 K, fluorescence was measured in a POLARstar Optima plate-reader (BMG LABTECH GmbH, Ortenberg, Germany) at 485/535 nm.

1.6.3 Fatty acid analysis

The composition of fatty acids was determined using the method by Lepage and Roy [25]. The total lipids, containing 0.01 % butylhydroxytoluene as antioxidant, were transesterified with acetyl chloride during 60 min at 373 K. Gas chromatography analysis was performed on a Shimadzu GCMS-QP2010 Plus gas chromatograph-mass spectrometer (Shimadzu, Kyoto, Japan). Fatty acid methyl ester peaks were identified by their elution pattern and relative retention times with respect to a reference mixture (GLC-744 Nu-Chek Prep. Inc., Elysian MN, USA). The results were expressed in relative amounts (molar percentage of total fatty acids).

2 Additional Results

2.1 Visual evidence of the effect of DHA on the aggregation of A_{2A} and D_2 protomers

Movie S1 clearly shows the striking preference for phospholipids containing DHA (i.e. SDPC) to surround A_{2A} and D_2 protomers. These shells foster quick protomer aggregation by improving their approach usually impaired when other lipids surround the protomers. In fact, this video clearly shows how a shell of SDPC surrounds A_{2A} and D_2 monomers virtually from the beginning of the simulation, and how the formed oligomers are still surrounded by SDPC molecules by the end of the simulation. Finally it shows the long time trend to form linear aggregate structures. This video corresponds to one of the 'Long initial' simulations. However, very similar behaviour is found in all other simulations.

In Movie S1, A_{2A} and D_2 protomers are depicted as pale blue spheres. Dark grey spheres correspond to all membrane lipids except for SDPC molecules, drawn as yellow spheres. Water, ions and anti-freezing particles were omitted for clarity. Note the periodic boundary conditions.

2.2 Preferred protein–protein interfaces

We used custom tcl and R scripts to analyse the frequency of each hetero- and homo-dimer interface formed in the CG-MD simulations (i.e. 'Long initial' and 'Homomers' systems in Table S1). The last 20 μ of each trajectory was used and all replicates for each system were pooled together. As shown in Fig. S1, the simulation data depicts two groups of helices preferentially involved in the A_{2A}–D_2 interface, namely transmembrane (TM) helices TM1 , TM2 and helix 8, or TM3, TM4 and TM5. Thus, both A_{2A} and D_2 monomers interact with other protomers through any of this two groups of helices, with TM5 (A_{2A}) – TM1 (D_2) being the most frequent interface. Similarly, both A_{2A} (Fig. S2) and D_2 (Fig. S3) homodimers form through TM1, TM2 and helix 8, or TM3, TM4 and TM5. However, while A_{2A} homomers tend to interact through helices TM3, TM4 and TM5 (Fig. S2), the most frequent interface in D_2 homomers involves TM1, TM2 and TM8 (Fig. S3).

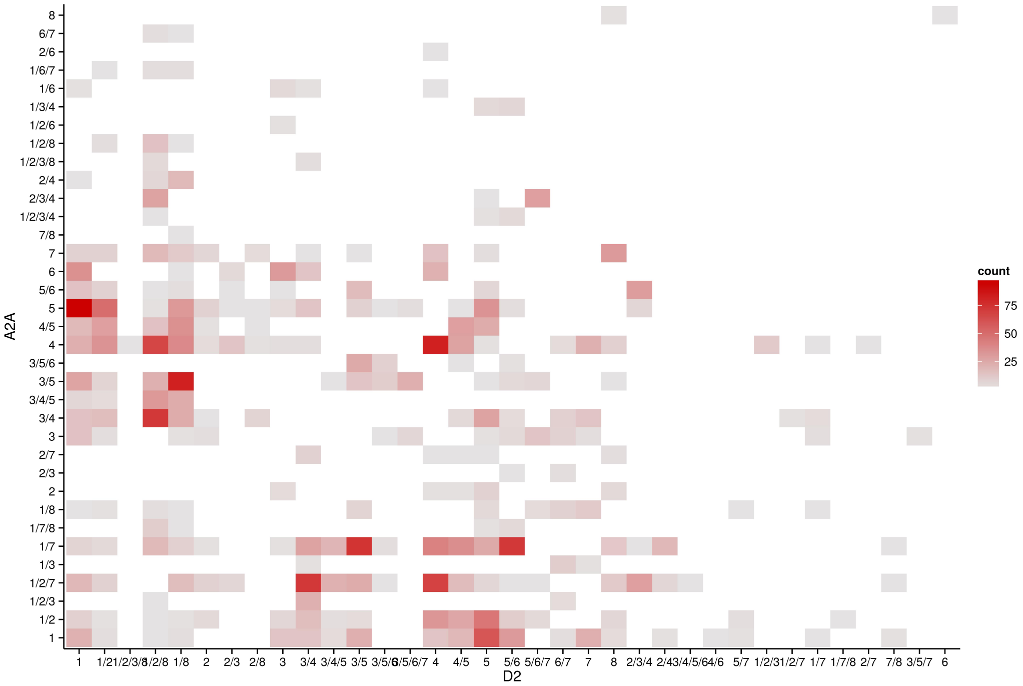

Figure S1: Most frequent interface of the A_{2A}–D_2 heterodimer from the pool of all initial trajectories ('Long initial' in Table S1). y and x axes represent, respectively, the A_{2A} and D_2 helices involved in each interface.

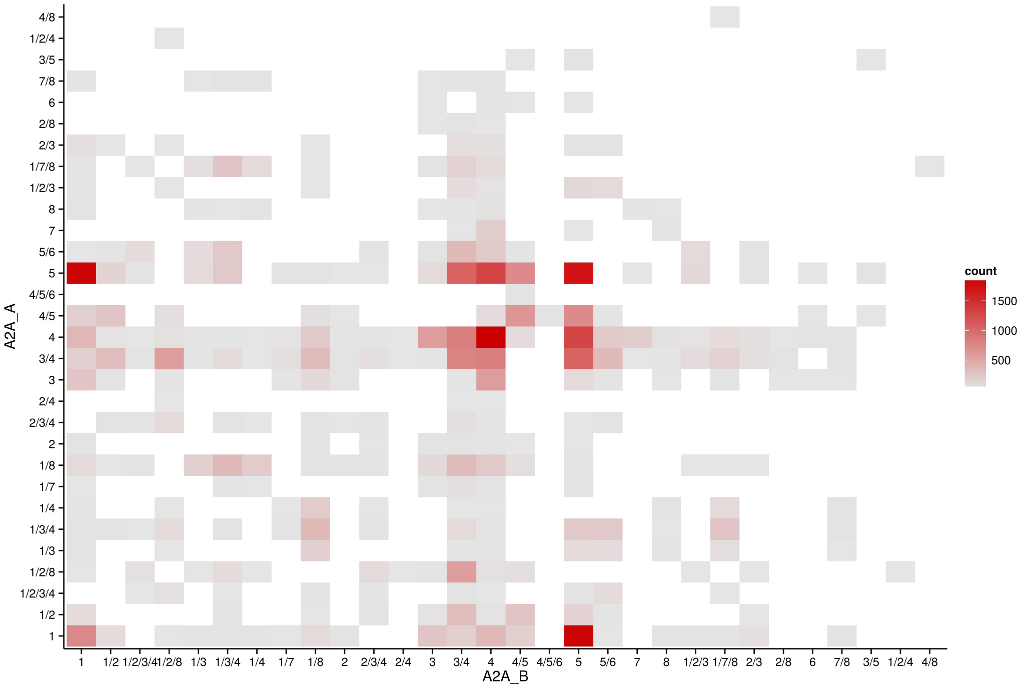

Figure S2: Most frequent interface of the A_{2A} homodimer from the pool of all A_{2A} homomer trajectories ('Homomers' in Table S1). y and x axes represent, respectively, the helices of each A_{2A} protomer involved in the interface.

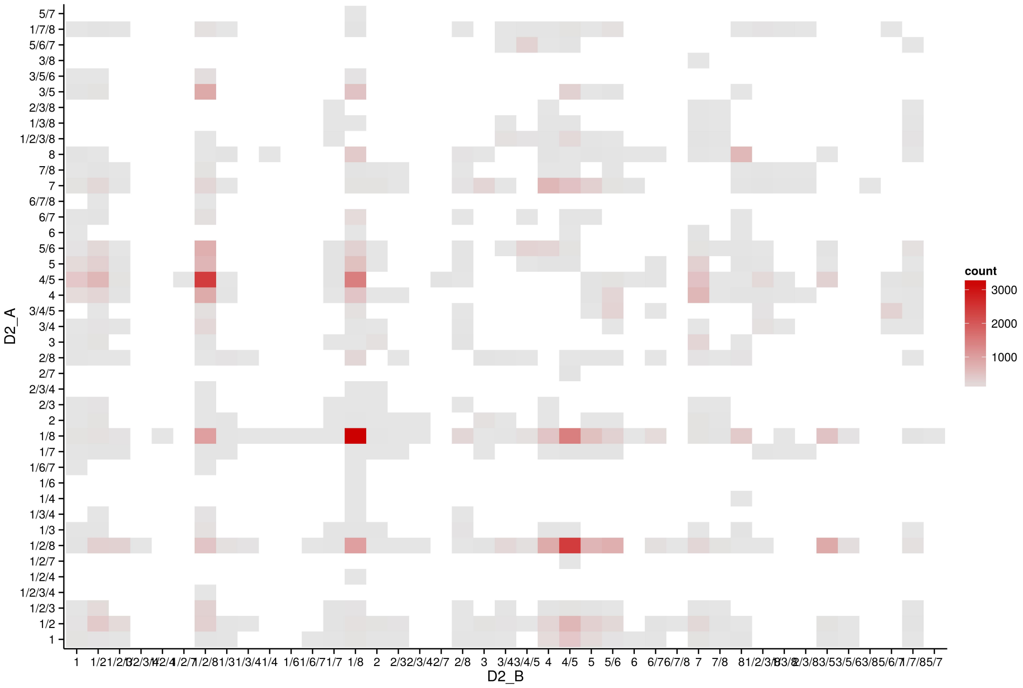

Figure S3: Most frequent interface of the D_2 homodimer from the pool of all D_2 homomer trajectories (‘Homomers’ in Table S1). y and x axes represent, respectively, the helices of each D_2 protomer involved in the interface.

2.3 Lipid RDFs around D_2 receptor in the CG-MD simulations

Radial distribution functions of lipids around A_{2A} monomers in one healthy-like and one diseased-like replica were shown in the main text. The corresponding plots are shown for D_2 in Fig. S4 for the same replicas. The tendency for the polyunsaturated fatty acid tails to solvate the receptor is in agreement with the results obtained for A_{2A}.

2.4 Lipid–protein contacts in the all-atom simulations

The relative proportions of atomic lipid–protein contacts over time are shown in Fig. S5. Details on the calculation of these ratios are given in section 1.4.1. Fig. S5a shows the evolution of 3 different contact ratios during the simulation, namely DOPC chains versus all saturated chains (i.e. DOPC / SAT), SDPC chains versus all saturated chains (i.e. SDPC /SAT) and DOPC chains versus SDPC chains (i.e. DOPC / SDPC). Fig. S5b displays the contact ratio of each SDPC chain versus all saturated chains (i.e. *sn*-1 / SAT and *sn*-2 /SAT). In all plots, SAT stands for all saturated chains. Coloured lines represent the smoothed average surrounded by the standard error.

A time-averaged volumetric map of DHA tails around the A_{2A} receptor during the 4 μs trajectory shows a general pattern of solvation by this fatty acid rather than specific interactions between DHA and certain protein helices (Fig. S6). In this trajectory, the area delimited by transmembrane helices (TM) TM3, TM4 and TM5 is less frequently occupied.

2.5 'Extended initial' healthy-like (DHA-high) CG-MD system

To study long-scale behaviour of protein oligomers, we extended the simulation of one of 'Long initial' CG-MD healthy-like systems from 60 to 260 μs (see Section 1.1.2 and Table S1 in this SI). The analysis of the last 20 μs of this 260 μs CG-MD simulation yielded a mean number of protein–protein contacts per protomer (i.e. the number of protomers that each protomer is in contact with) of 1.71. Snapshots of this simulation at 65, 130, 195 and 260 μs are shown in Fig. S7. These snapshots show how the structure of the oligomer evolves until all protomers are participating in protein–protein contacts. These interactions seem strong and the structure of this protein aggregate very stable. Once receptors come into close contact, they do not seem to separate thus it seems unlikely that PUFAs would play a major role on the equilibrium binding mode of this oligomer. In addition, dissociation events are rare in the probed time scale. Therefore, the equilibrium effect of PUFAs is most likely negligible, in agreement with our BRET experiments.

2.6 Validation of the influence of initial protein arrangement on the nature of protein–protein interactions

To validate the influence of the initial placement of protein monomers on the nature of the protein–protein interactions (i.e. fraction of heteromers versus homomers), we performed simulations using different initial arrangements of protein monomers ('Validation' in Table S1 and Table S2b). Specifically, we initially placed protein monomers so that each receptor type occupied one half of the simulation box (see Fig. S8a), and ran 3 replicas of healthy- and diseased-like systems for 120 μs. As shown by snapshots taken at the end of these simulations (see Fig. S8b–d), the initial arrangement of protein monomers indeed drives the heteromer/homomer ratio measured at the end of the simulation. To be more precise, in these simulations only 30 % of the complexes, in average, corresponds to A_{2A}–D_2 heteromers. Similarly, the same tendency is observed in simulations using monomers of the same receptor type (see Figs. S11 and S12).

Therefore, the initial arrangement of proteins determines the nature of the final aggregates and the interaction between protomers in our simulations, however, the enhancing effect of DHA on protein aggregation is preserved across each validation set.

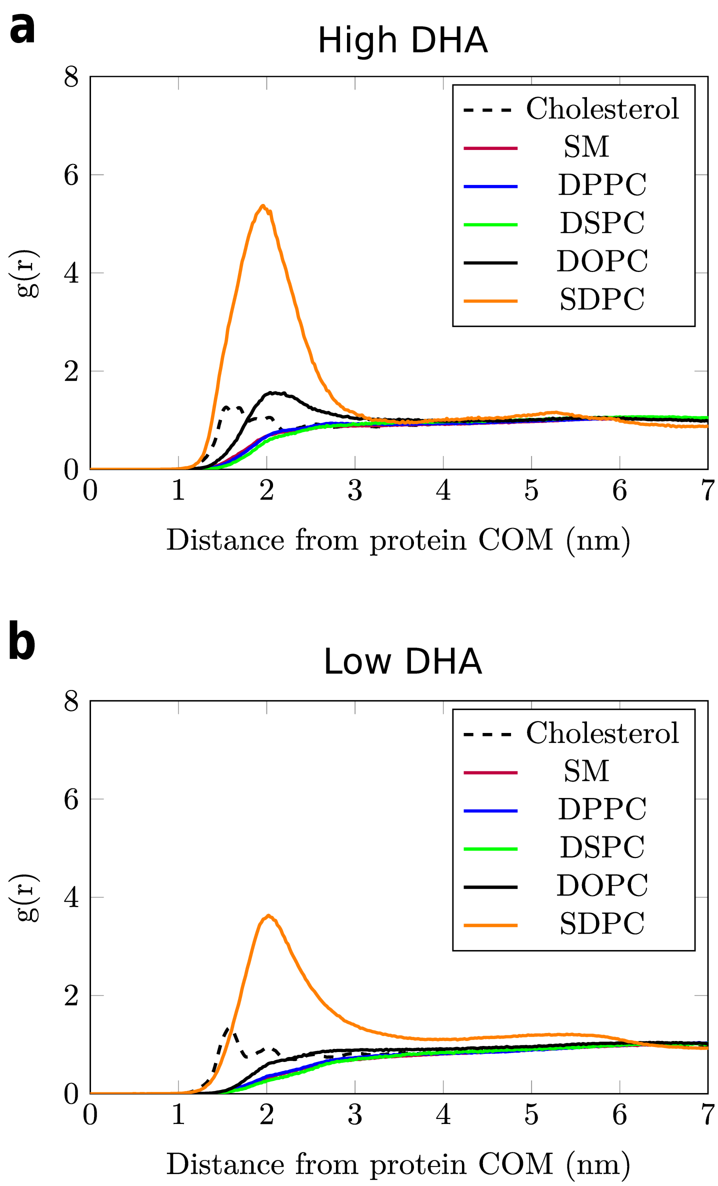

Figure S4: Probability density (i.e. radial distribution function, $g(r)$) of lipids around the center of mass (COM) of the D_2 receptor embedded in healthy- (high DHA, **(a)**) and diseased-like (low DHA, **(b)**) model membranes in CG-MD simulations. y axis represent $g(r)$ (arbitrary units) and x axis the distance to the protein COM in nm. The radial distribution function of SM heavily overlaps with the rest of saturated lipids (i.e. DPPC and DSPC).

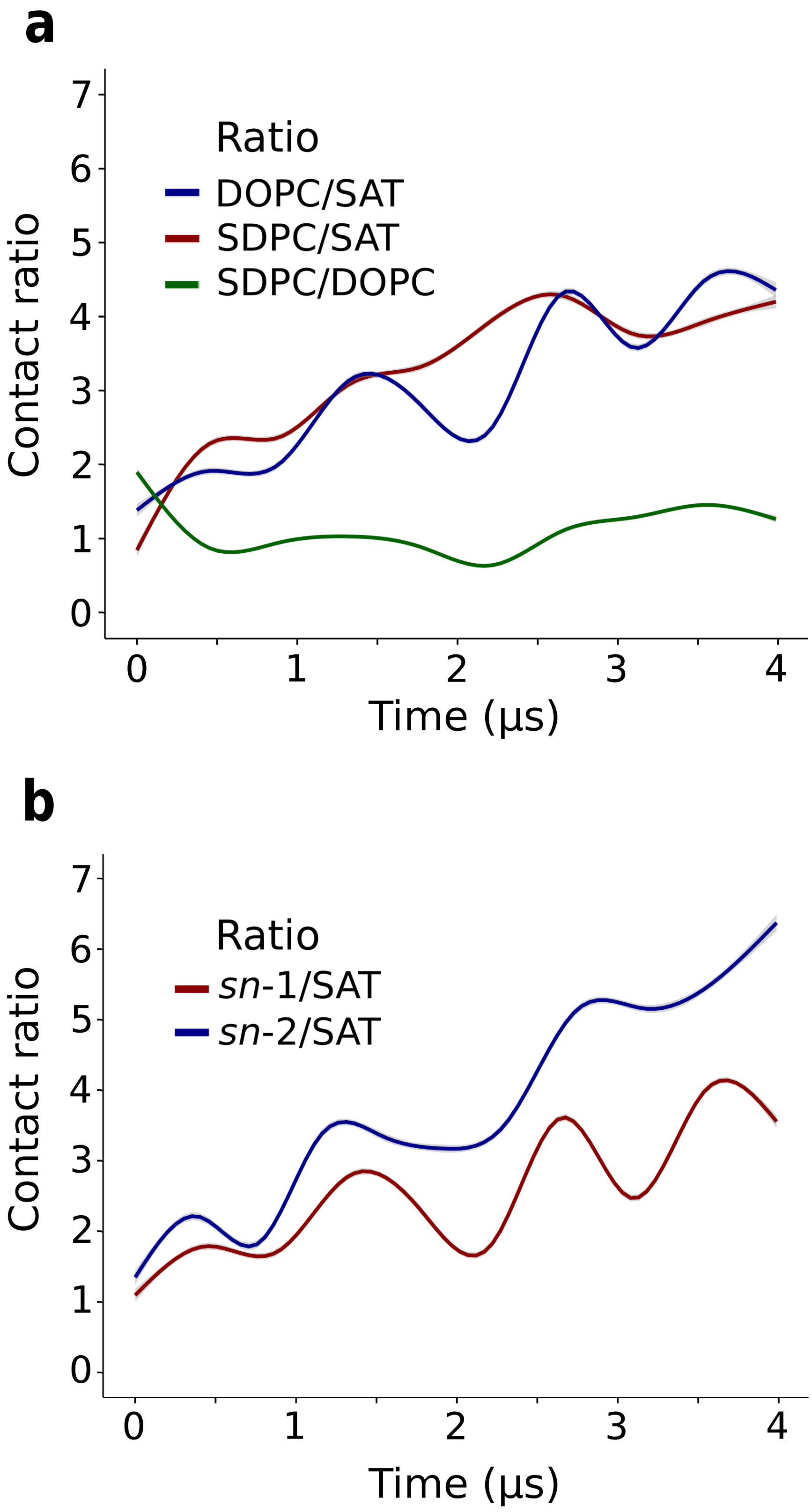

Figure S5: Evolution of lipid–protein contacts during the all-atom simulation, see section 1.4.1 for details.

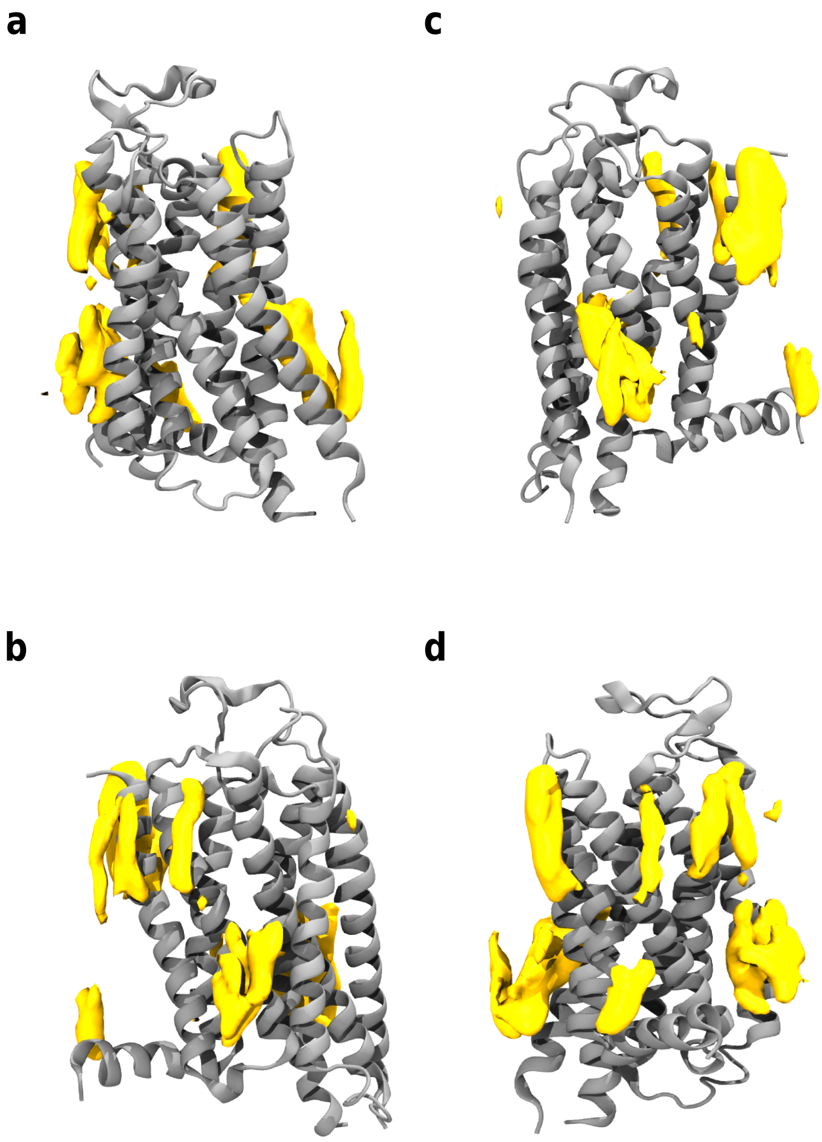

Figure S6: Time-averaged 3D volumetric map of DHA tails around the A_{2A} receptor (grey cartoons) laterally viewed from the membrane bilayer plane at four different angles (Transmembrane helices (TM) left to right): **(a)** TM3, TM4 and TM5; **(b)** TM8, TM1, TM2, TM3, TM4 and TM5; **(c)** TM5, TM6, TM7, and TM8; and **(d)** TM6, TM7, TM8, TM1, and TM2. In all figures, top is extracellular and bottom intracellular. The density volume of DHA is depicted in yellow. The rest of lipids, ions and water molecules were omitted for clarity. Default parameters plus an isovalue of 0.2 were set in VMD 1.9[17] to visualize DHA volumetric data as isosurface.

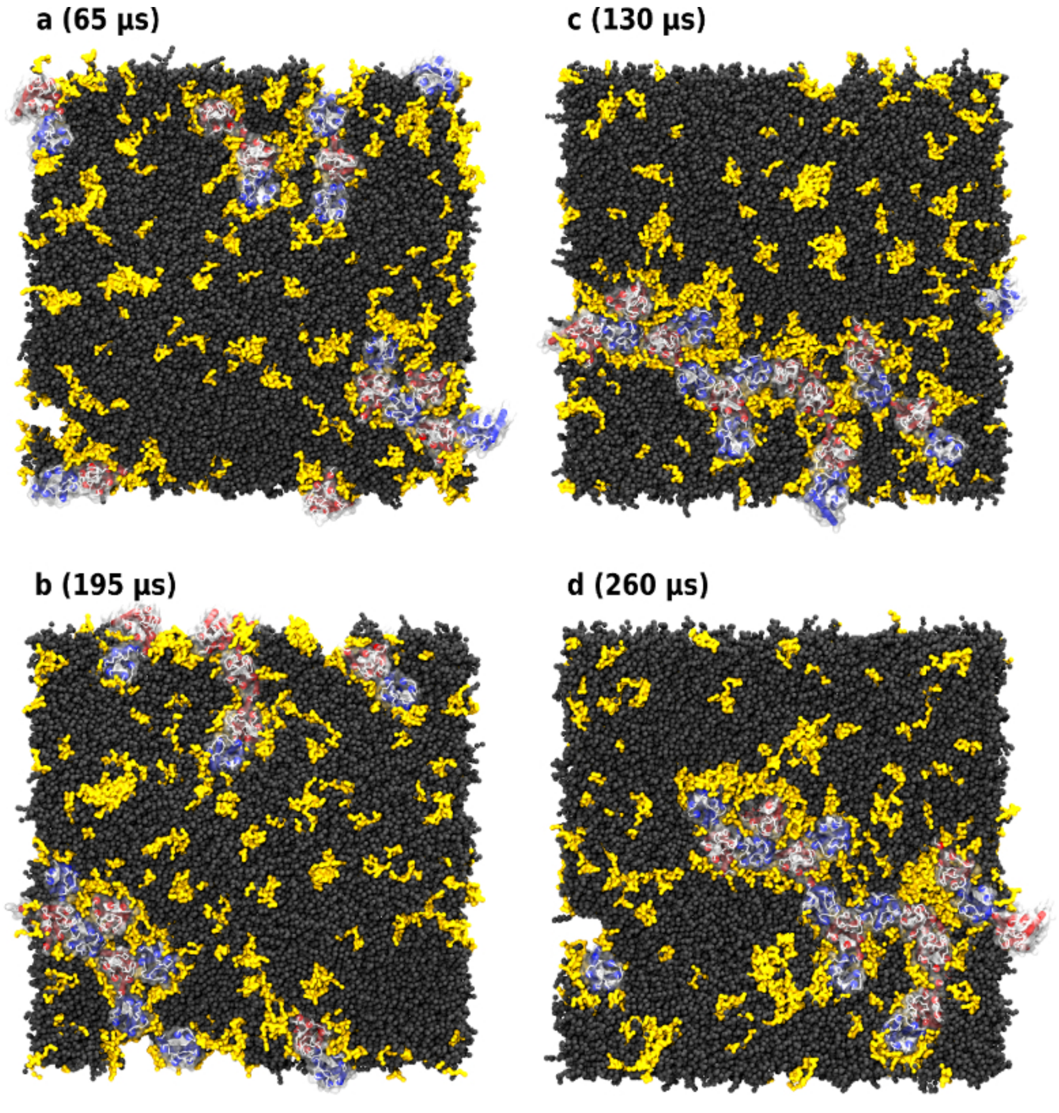

Figure S7: Long-scale behaviour of protein oligomers in the 'Extended initial' simulation (see Table S1). A_{2A} and D_2 helices are depicted in red and blue cartoons, respectively. Protomers are surrounded by a white transparent surface. Dark grey spheres correspond to a van der Waals representation of all membrane lipids except for SDPC molecules, depicted in yellow surface. Water molecules, ions and anti-freezing particles were omitted for clarity. Note the periodic boundary conditions.

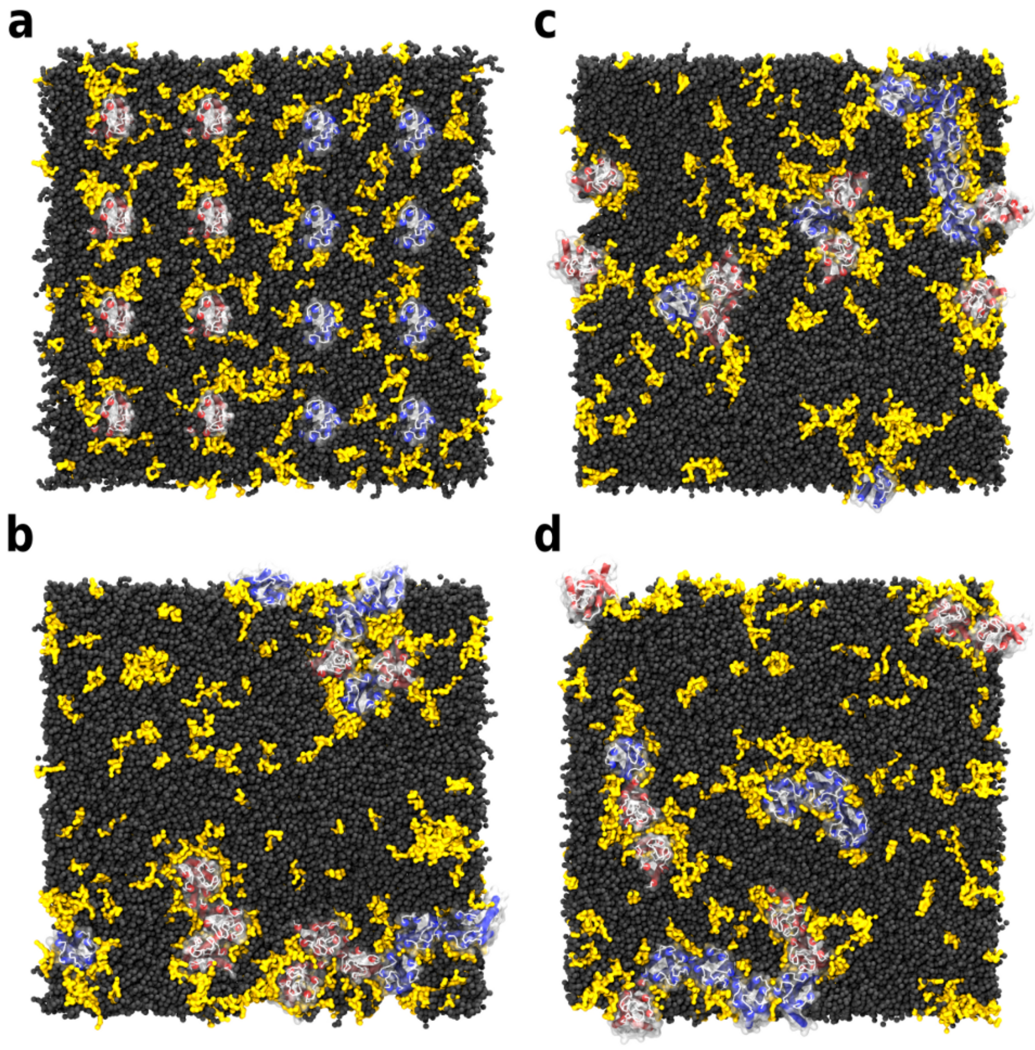

Figure S8: Initial (**a**) and three final arrangements (**b–d**) of protein oligomers in the three replicas of healthy-like system of the 'Validation' simulation set (see Table S1). A_{2A} and D_2 helices are depicted in red and blue cartoons, respectively. Protomers are surrounded by a white transparent surface. Dark grey spheres correspond to a van der Waals representation of all membrane lipids except for SDPC molecules, depicted in yellow surface. Water molecules, ions and anti-freezing particles were omitted for clarity. Note the periodic boundary conditions.

2.7 Protein–protein contacts in the CG-MD simulations

The time evolution of protein–protein contacts is shown in the main text for the three replicas representing both healthy-like (DHA-high) and diseased-like (DHA-low) membranes. The aggregation plots for the additional, shorter (16 μs) simulations mentioned in the main text are shown in Fig. S9. Five replicas of both lipid compositions were simulated. These plots confirm that receptor aggregation is more rapid in the healthy-like membrane where differences caused by the lipid composition become significant between 4–12 μs.

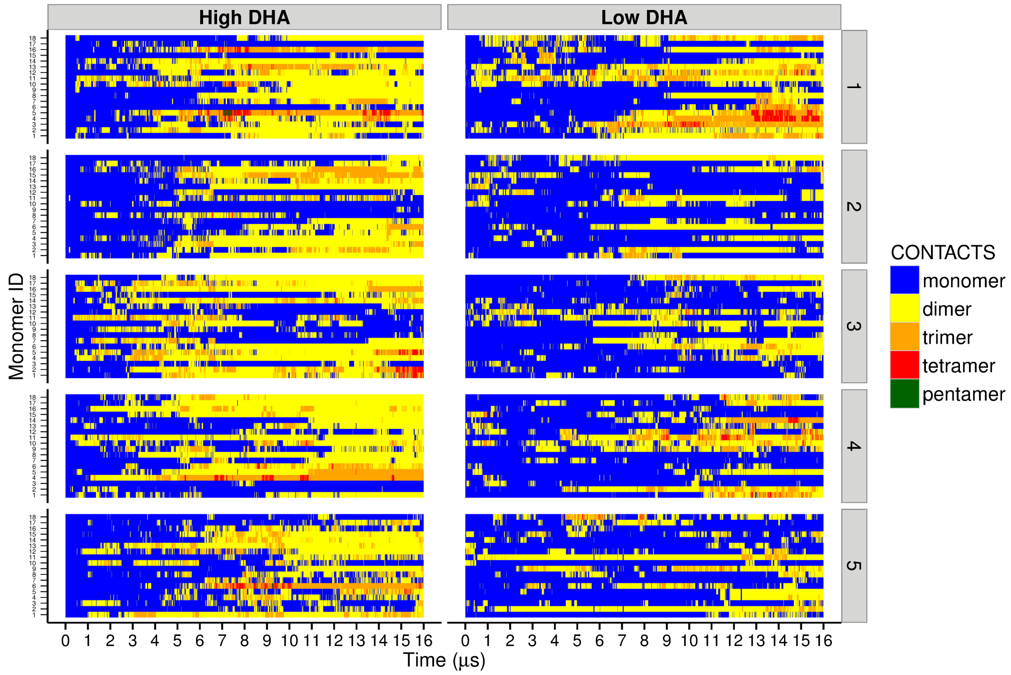

Figure S9: Aggregation patterns of the 5 replicas for both compositions simulated for 16 μs ('Short initial' in Table S1). Healthy-like (i.e. DHA-high, left) and diseased-like (DHA-low, right) systems where each cell represents one replicate. Each row in the y axis represents one GPCR protomer and time extends along the x axis.

Additional confirmation for the effect of DHA on oligomerisation was obtained by considering a different initial arrangement of the protomers. Namely, D_2 and A_{2A} protomers were initially placed on different sides of the membrane ('Validation' in Table S1). The aggregation patterns for each of the three replicas of both membrane compositions are shown in Fig. S10. Even though this initial arrangement of receptors affects the ratio of homomers and heteromers, the effect of DHA on increasing aggregation kinetics remains nevertheless.

The time evolution of protein–protein contacts for the homomerisation of both A_{2A} and D_2 protomers ('Homomers' in Table S1) is shown in Figs. S11 and S12, respectively. While the kinetic effect exerted by DHA is again present across all systems, the overall aggregation kinetic pattern is different between both protein species.

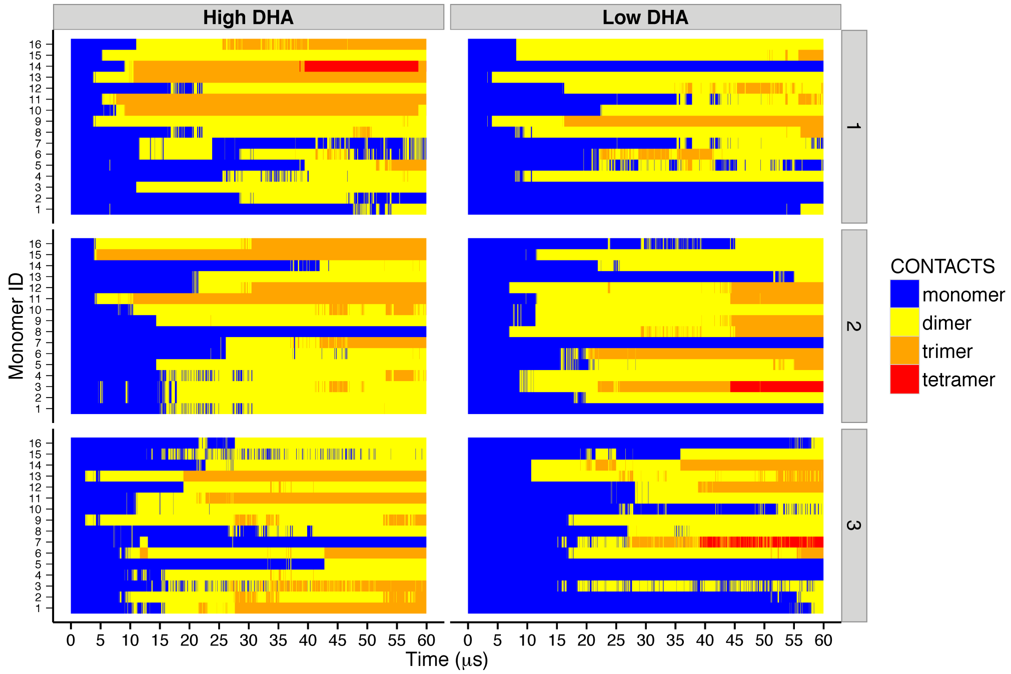

Figure S10: Time-dependence of protein aggregation in 'Validation' CG-MD simulations starting from different initial arrangements of protomers. Healthy-like (i.e. DHA-high, left) and diseased-like (DHA-low, right) systems where each cell represents one replicate. Each row in the y axis represents one A_{2A} or D_2 protomer.

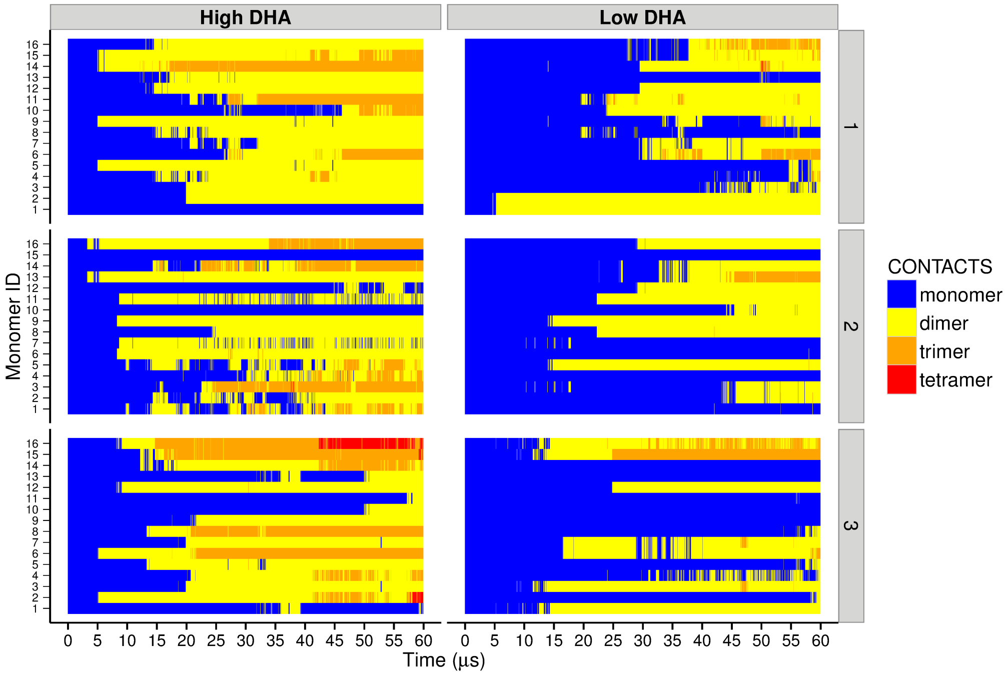

Figure S11: Time-dependence of protein homomerisation in 'Homomers' CG-MD simulations of A_{2A} receptors embedded in healthy-like (i.e. DHA-high, left) and diseased-like (DHA-low, right) membranes. Each cell represents one replicate. Each row in the y axis stands for one A_{2A} protomer.

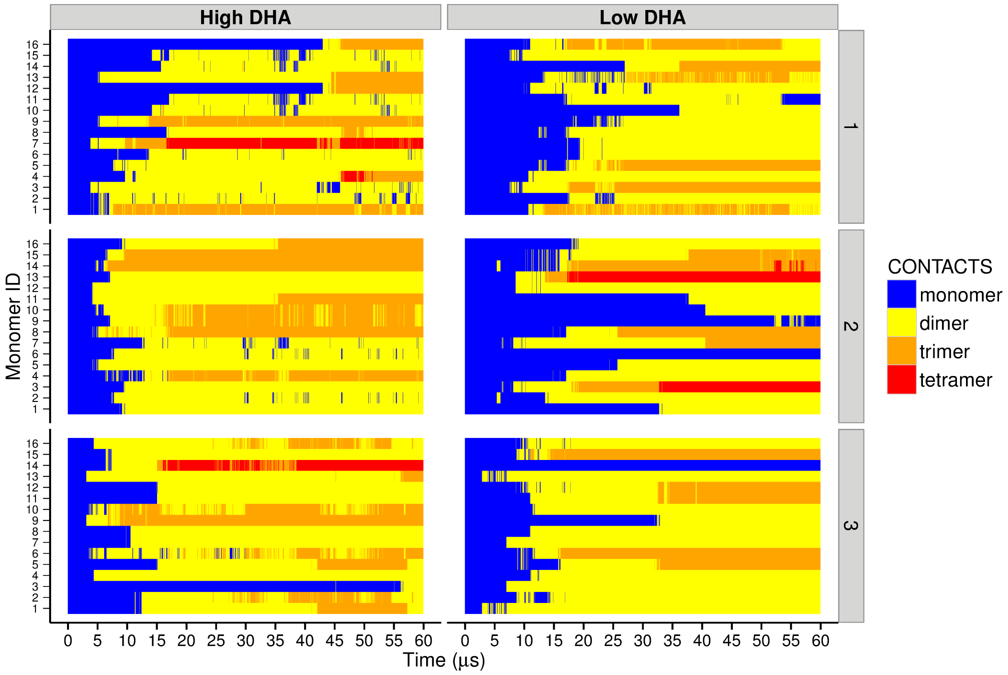

Figure S12: Time-dependence of protein homomerisation in 'Homomers' CG-MD simulations of D_2 receptors embedded in healthy-like (i.e. DHA-high, left) and diseased-like (DHA-low, right) membranes. Each cell represents one replicate. Each row in the y axis stands for one D_2 protomer.

2.8 Receptor and lipid diffusion in the 'Single-protein' CG-MD system

Protein diffusion coefficients could not be extracted from the simulations of the CG-MD multi-protein systems due to the rapid oligomerisation. This oligomerisation slows down diffusion significantly and therefore long time data on individual receptor motion, required for the proper determination of the diffusion coefficients, cannot be obtained. Instead, we simulated individual A_{2A} and D_2 receptors in both healthy-like and diseased-like membranes ('Single-protein' in Table S1). The time-averaged mean squared displacement (MSD) curves of the proteins are shown in Fig. S13, whereas the extracted diffusion coefficients are given in the main text and in Table S5. Lipid diffusion coefficients, extracted from these 'Single-protein' systems, are also given in Table S5.

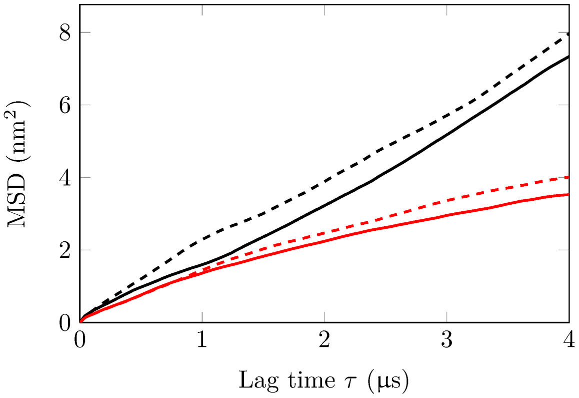

Figure S13: Mean squared displacement (MSD) of individual proteins in the 'Single-protein' CG-MD simulations. Data show the average MSD of proteins embedded in healthy-like (black curve) and diseased-like (red curve) membrane environments for A_{2A} (solid) and D_2 (dashed) receptors.

Table S5: Diffusion coefficients extracted for different A_{2A} and D_2 receptors as well as lipid components from the 'Single-protein' CG-MD simulations. Values are reported in 10^{-8} cm^2/s $\pm$ the error estimate.

	Healthy, A_{2A}	Healthy, D_2	Diseased, A_{2A}	Diseased, D_2
A_{2A}	0.48 ± 0.13	–	0.18 ± 0.06	–
D_2	–	0.46 ± 0.1	–	0.22 ± 0.06
Cholesterol	5.1 ± 0.1	4.9 ± 0.6	3.5 ± 0.1	3.5 ± 0.4
SM	4.2 ± 0.2	3.6 ± 0.2	3.2 ± 0.0	3.5 ± 0.1
DPPC	3.6 ± 0.6	3.7 ± 0.9	3.4 ± 0.4	3.9 ± 0.3
DSPC	3.7 ± 0.3	3.2 ± 1.1	3.2 ± 0.5	3.9 ± 0.6
DOPC	4.8 ± 0.8	4.6 ± 0.2	3.5 ± 0.0	4.3 ± 0.5
SDPC	3.8 ± 0.1	4.4 ± 0.1	2.4 ± 0.7	2.9 ± 0.6

2.9 Rotational diffusion of the receptors in the 'Single-protein' CG-MD system

The decay of the rotational autocorrelation function describes how fast the molecule in question rotates around its own axis. Therefore, the rotational autocorrelation functions of A_{2A} and D_2 receptors with respect to the membrane normal in the healthy-like and diseased-like membrane environments reveal their abilities to sample different oligomerisation interfaces. These rotational autocorrelation functions are shown in Fig. S14 for short and long times. The second order Lagrange polynomial is used to calculate the autocorrelation function.

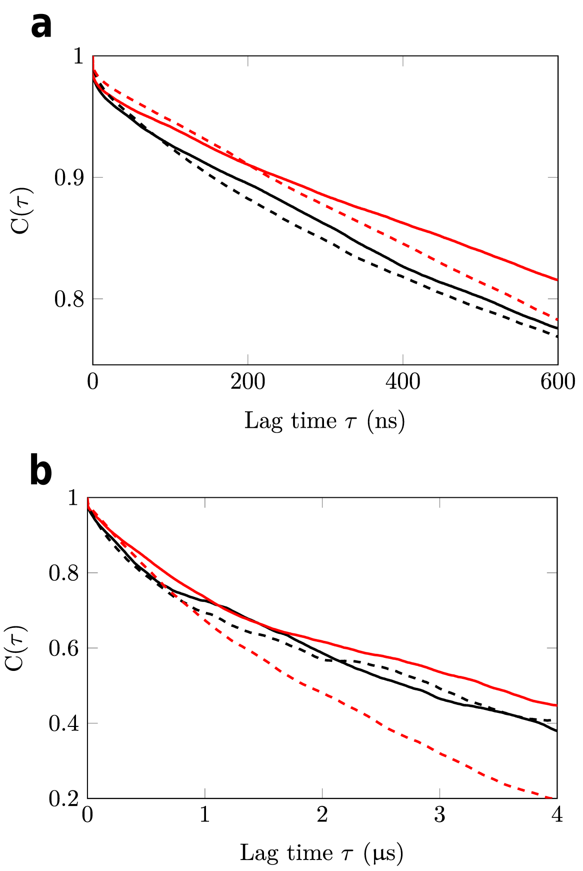

Figure S14: Rotational autocorrelation of protein motion in the 'Single-protein' CG-MD simulations over short **(a)** and long **(b)** times. Second order Lagrange polynomial is employed as the rotational autocorrelation function, $C(\tau)$, and plotted in the y axis. Lag time τ, in μs, extends along the x axis. Solid and dashed lines correspond to A_{2A} and D_2 whereas black and red stand for healthy- and diseased-like systems, respectively.

2.10 Lipid diffusion in the crowded multi-protein CG-MD system

The lateral diffusion coefficients of lipids in the multi-protein 'Long initial' CG-MD systems reported in the main text were extracted from the mean squared displacement (MSD) curves shown in Fig. S15.

2.11 Aggregation plots for the 'Very high DHA' control system

Fig. S16 shows the aggregation patterns for each of the 3 replicas of the control system containing very high amounts of DHA ('Very high DHA' system in Table S1). In these systems 62.5% of the phospholipids are SDPC. Therefore, DHA fatty acid tails accounts for 31% of the total fatty acid tails. The detailed composition of this system is given in Table S3b.

2.12 Partial phase separation in the healthy-like 'Protein-free' CG-MD membrane

The snapshots corresponding to 0, 13.3, 26.7 and 40 μs of simulation of the 'Protein-free' CG-MD system of a healthy-like lipid composition (see Table S1) are shown in Fig. S17. These snapshots suggest that the membrane undergoes an at least partial phase separation into DHA-enriched and DHA-depleted domains, yet this separation is not complete.

This observation is backed up by the contact fraction, calculated following Ref 26, obtained from

$$f_{\mathrm{US-S}} = \frac{c_{\mathrm{US-S}}}{c_{\mathrm{US-US}} + c_{\mathrm{US-S}}}, \qquad (2)$$

where $c_{\mathrm{US-S}}$ stands for the number of contacts between unsaturated (DOPC and SDPC) and saturated (DPPC, DSPC, sphingomyelin) lipids and $c_{\mathrm{US-US}}$ corresponds to the number of contacts between the unsaturated lipids. A contact was registered if the distance between the phosphate group beads (bead PO4 in Martini force field) was smaller than 1.1 nm.

The time evolution of $f_{\mathrm{US-S}}$ is shown in Fig. S18. Indeed, the system undergoes phase separation at some level until approximately 25 μs, after which demixing no longer takes place. As saturated lipids make up for 64% of the non-cholesterol lipids in the membrane, ideal mixing would result in a value of 0.64, whereas complete phase separation would result in a really small value tending toward 0. The obtained values are clearly below 0.64 but much larger than zero suggesting that separation occurs but is indeed not perfect.

2.13 Effect of the presence of DHA shells on protein dimerisation

Characteristic examples of the time evolution of the receptor–receptor distance during an oligomerisation event are shown in Fig. S19. These curves show how receptors without a DHA coating in the diseased-like system (DHA low) can sample fairly small inter-protein distances (< 5 nm) without rapidly aggregating. Receptors in the healthy-like system (DHA high), however, are quickly drawn together as soon as their DHA shell come into contact at inter-protein distances of $\sim$6 nm.

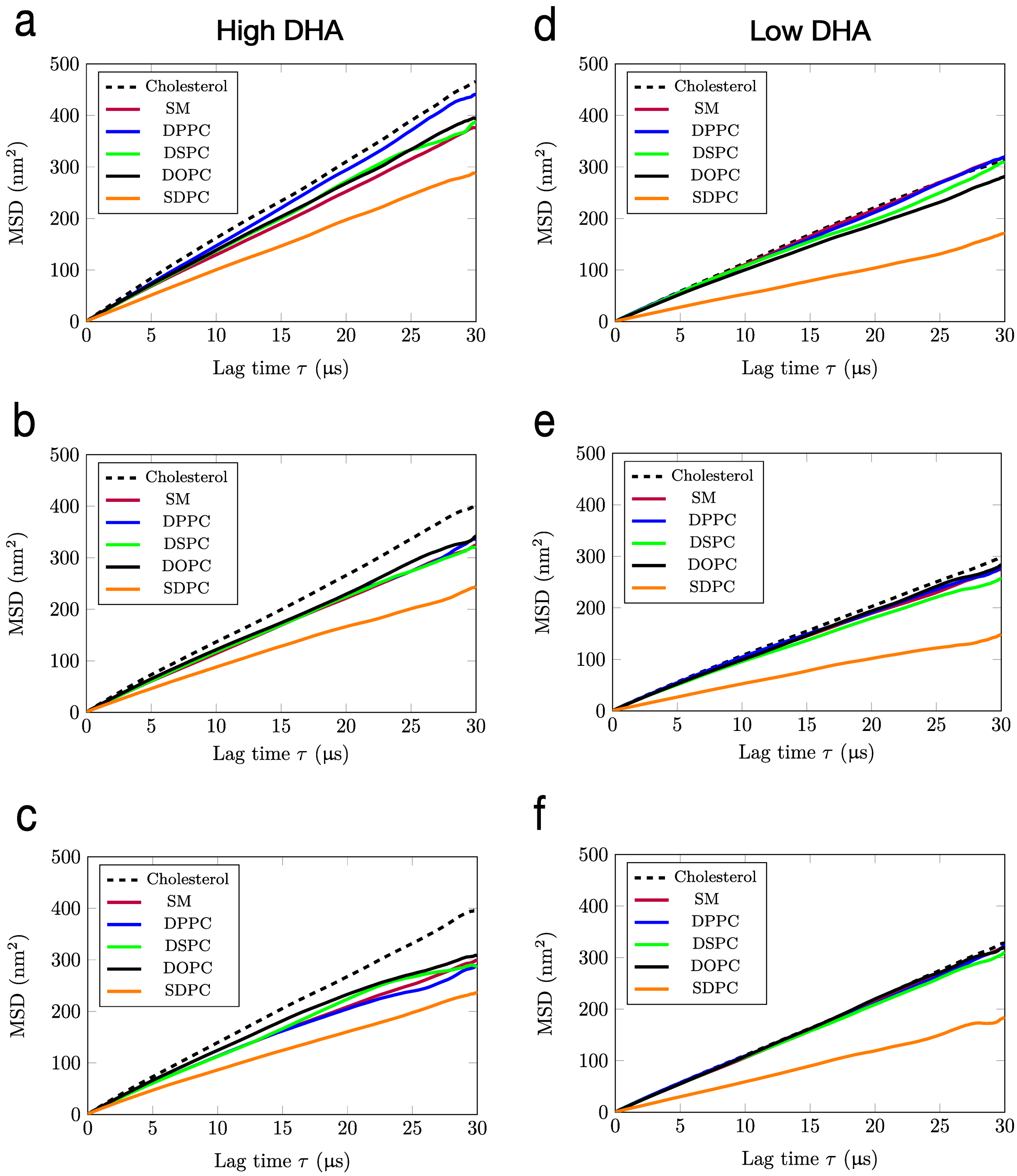

Figure S15: Mean squared displacement in the 'Long initial' CG-MD simulations is plotted for each lipid species in healthy- (DHA-high, **(a–c)**) and diseased-like systems (DHA-low, **(d–f)**). Each cell represents one replicate.

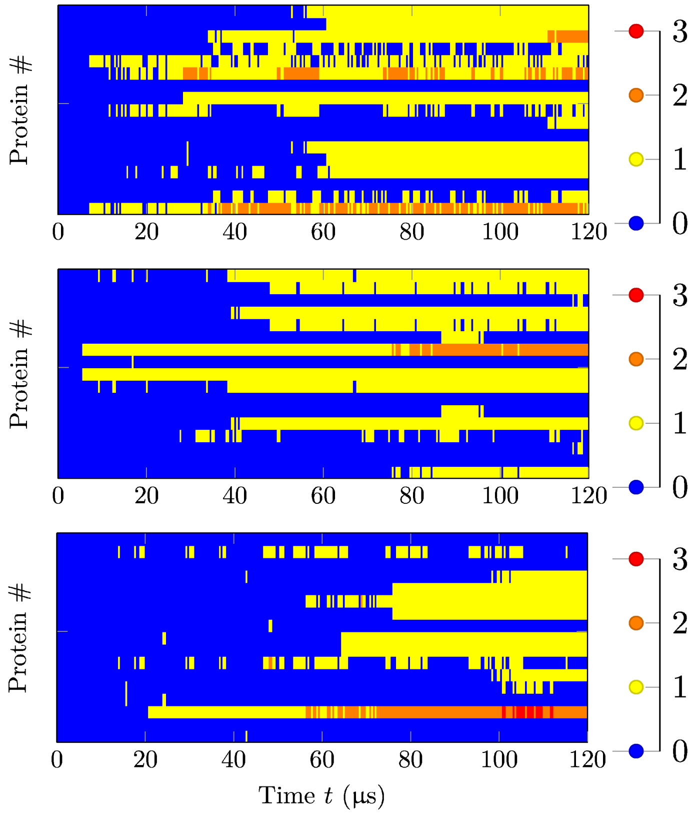

Figure S16: Aggregation patterns for the three replicas of the 'Very high DHA' system. Each horizontal bar represents one GPCR protomer. The colour code reflects the number of contacts per protomer.

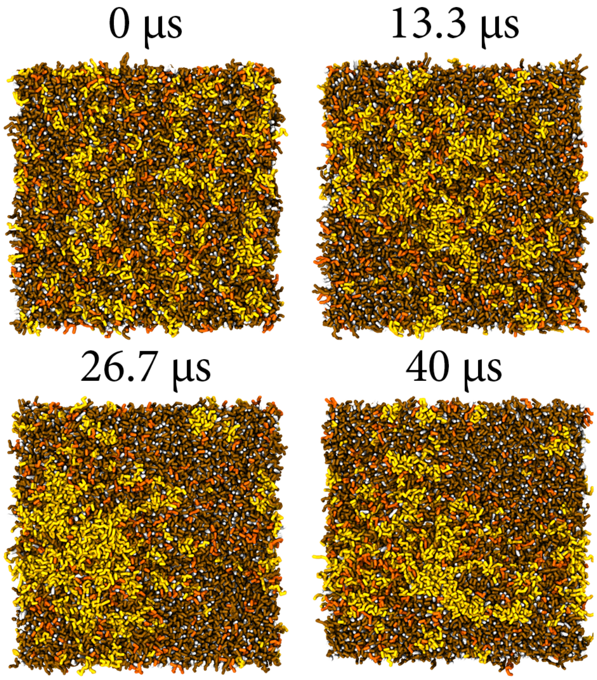

Figure S17: Snapshots showing the time evolution of lipid demixing in a healthy-like 'Protein-free' CG-MD system, i.e. system without proteins. All saturated lipids (DPPC, DSPC and sphingomyelin) are shown in brown, the monounsaturated phospholipid DOPC is depicted in orange and the DHA-containing phospholipid SDPC is drawn in yellow. Cholesterol is shown in white.

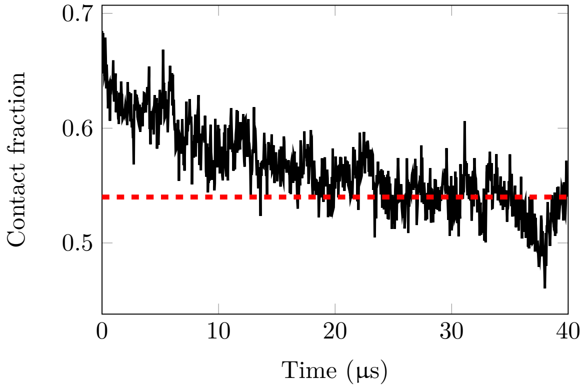

Figure S18: Time evolution of the contact fraction between the saturated and unsaturated lipids defined by Eq. (2) in a healthy-like 'Protein-free' CG-MD system. The dashed red line shows the potential equilibrium value at 0.54.

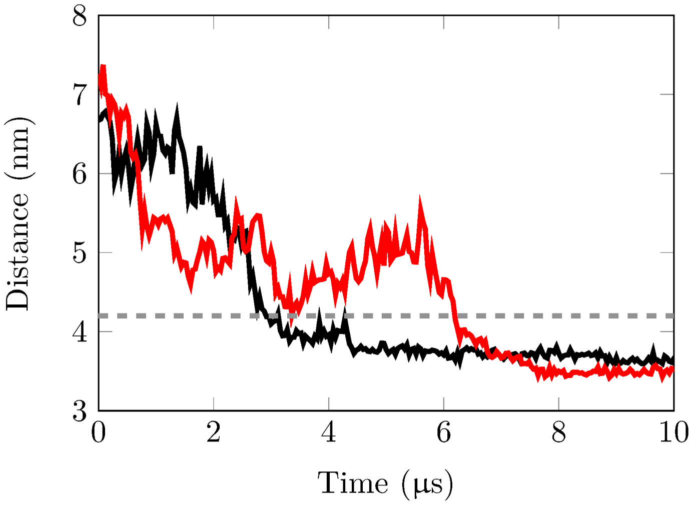

Figure S19: Characteristic examples of protein dimerisation events taken from a healthy-like (DHA-high, black) and diseased-like (DHA-low, red) in ‘Long initial’ CG-MD systems. The value of 4.2 nm, employed as a benchmark for a protein–protein contact, is shown with a dashed grey line.

2.14 Matching computational and experimental levels of DHA

One might assume that the ambiguous nature of the role of DHA on the oligomerisation, arises from the differences in DHA content between computer simulations and BRET experiments. DHA-high and DHA-low cell models were generated for our BRET experiments by feeding the HEK-293T cells with triglyceride fish oil. However, in this experiments, we could not reach the same amount of DHA as modelled in our initial computer simulations (see Tables S2 and S3). To make sure this mismatch does not significantly bias our conclusions, we performed a final set of MD simulations mimicking the exact DHA concentration used in the BRET experiments (see Table S1 and Table S2d). In order to reach DHA experimental levels we only modified the amount of SDPC and DSPC present in the initial simulations. The results from these simulations (Fig. S20) confirm that the levels of DHA employed in our BRET experiments are sufficient to enhance protein oligomerisation kinetics of GPCRs in the probed time scale.

The aggregation plots for the systems with the DHA concentrations matching the ones employed in the BRET experiments, i.e. 1% and 6.5%, are shown in Fig. S20. The detailed composition of this system is given in Table S2d.

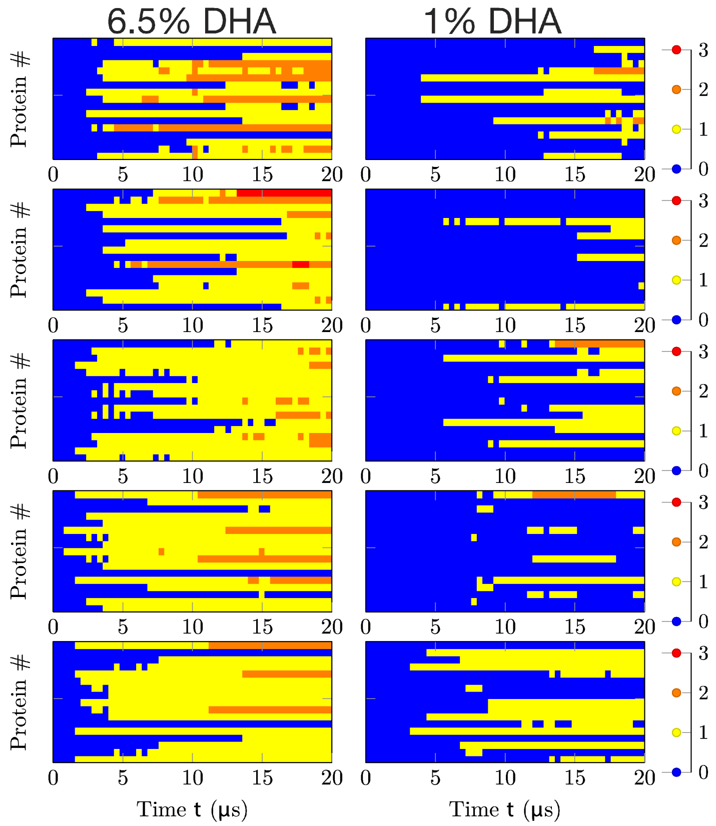

Figure S20: Aggregation patterns for the five short replicas of the systems whose DHA contents match those measured for the DHA-enriched and DHA-depleted cells employed in BRET experiments ('BRET composition' in Table S1). The colour code reflects the number of contacts per protomer.

2.15 Validation of the DHA effect by tweaking the Martini force field

One limitation of the CG-MD models employed here is the stable nature of the protein complexes formed during the simulations. In our simulations, the formation of protein–protein contacts seems to be a highly irreversible process at least within the probed time scales (i.e. hundreds of μs). This effect is consistent with similar studies on the aggregation of other membrane proteins[27–29]. Since all protein monomers will eventually engage in protein–protein interactions, the effect of DHA on the oligomerisation state in equilibrium is likely negligible. Likewise, the strong oligomerisation tendency of receptors could indicate a force field overestimation of protein–protein interactions, as previously shown for water-soluble proteins[30]. Thus, recent studies using Martini have shown that this force field overestimates the stability of membrane protein dimers when compared to experimental results[31] or all-atom force fields[32]. Conversely, certain studies shows that Martini force field is able to yield results in better agreement with experiments than some atomistic force fields[33]. Since the CG-MD simulations in this paper were run using version 2.1 of the Martini force field, we did not benefit from the improvements made to the latest version (2.2) of the protein force field[34].

However, it has been suggested[30], that protein aggregation behaviour in Martini would improve by scaling down the ε values of the interactions between protein beads. To investigate the effect of tweaking ε values in our simulations, we run two extra simulations (healthy-like and diseased-like membrane compositions) using version 2.2 of the Martini and scaling down all protein–protein interactions by 10%. The results from these simulations show that scaling down ε values significantly improves the sampling of potential protein dimerisation interfaces. Nevertheless, the effect of DHA on fostering oligomerisation is still clearly visible (see Fig. S21), in agreement with the results obtained using the unmodified version of the force field.

The aggregation plots for the systems in which version 2.2 of the Martini force field[34] was used together with a 10% reduction in the protein–protein interactions are shown in Fig. S21. Data is shown for the initial healthy-like and diseased-like membrane compositions.

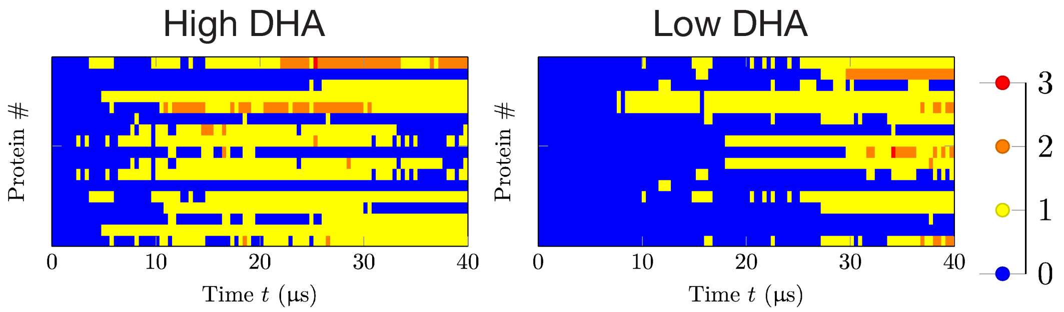

Figure S21: Aggregation patterns for the healthy-like (left) and diseased-like (right) membrane compositions simulated with version 2.2 of the Martini protein force field[34] and with the protein–protein interactions scaled down by 10%. The colour code reflects the number of contacts per protomer.

2.16 DHA is successfully incorporated into HEK-293T cells upon DHA fatty acid supplementation

Prior to DHA incorporation, we determined the fatty acid content of parental HEK-293T cells grown under standard conditions. Under these basal conditions, the cells showed a low relative content of DHA: 0.99 ± 0.03 % of total fatty acids (see Fig. S22). To obtain a cell culture with a higher level of DHA, the HEK-293T cells were supplemented with increasing concentrations of DHA. Interestingly, we observed saturable dose-dependent incorporation of DHA into the membrane of HEK-293T cells with a maximum dose achieved at 200 µM of DHA, yielding a relative DHA content of 6.49 ± 0.32 % of total fatty acids (see Fig. S22). While this content is below the level of DHA found in normal human brains[10], cells supplemented with the maximum dose of DHA still show more than 6-fold higher DHA than cells grown in basal conditions. Therefore, we established non-treated cells and cells treated with 200 µM DHA as representatives of DHA-low and DHA-enriched models, respectively.

Remarkably, we did not observe significant DHA-mediated cytotoxicity at any dose tested: cell viability at 200 µM DHA was 89.5 ± 8.2 % compared to untreated cells (p=0.3045, Student's *t* test, n=3). With regard to fatty acid profile, while levels of saturated fatty acids remained constant, treatment with DHA induced an increase of PUFA and ω-3 series along with a progressive decrease in monounsaturated and ω-6 series, resulting in a five-fold decrease in the ω-6 / ω-3 ratio (see Fig. S22).

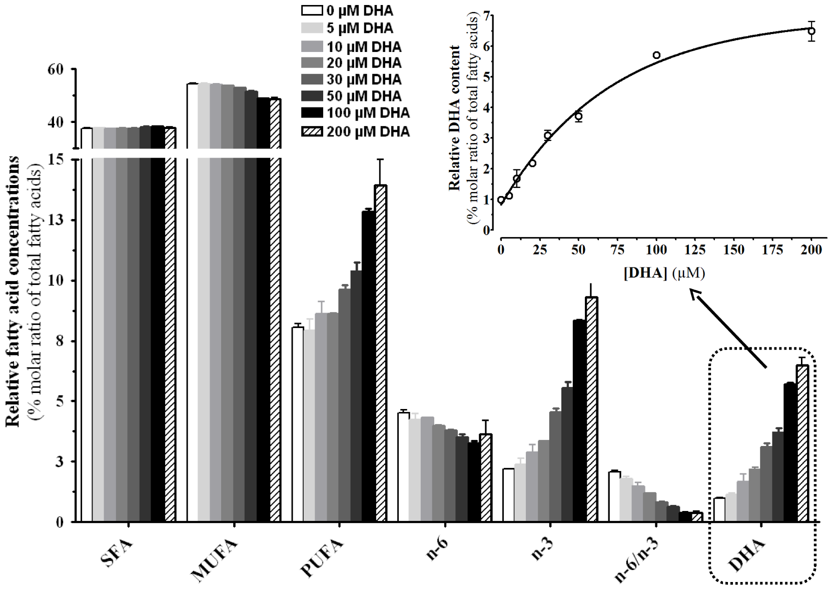

Figure S22: Effect of DHA treatment in the fatty acids content of HEK cells. HEK-293T cells were incubated with increasing concentrations of DHA (22:6n-3) during 48 h. The effect of increasing doses of DHA in the fatty acid profile was measured by gas chromatography. The specific dose-dependent DHA incorporation is shown (inset panel). The fatty acid content is expressed as the relative molar ratio in percentage. Data shown are the mean ± SEM of three determinations. SFA, MUFA, PUFA, n-6 and n-3 labels stand for saturated fatty acids, monounsaturated fatty acids, polyunsaturated fatty acids, ω-6 PUFA and ω-3 PUFA, respectively

2.17 A note regarding lateral diffusion coefficients: comparison with experimental data

The diffusion coefficients calculated in this manuscript for proteins and lipids agree reasonably well with experimental values. However, the direct comparison is unfortunately not feasible due to the complexity of the employed lipid mixture as well as the presence of proteins, which is known to hinder the diffusion of both lipids and proteins. Here we compare the calculated diffusion coefficients to those obtained in model lipid membranes and cells.

In their work on membranes containing bacteriorhodopsin (BR), Peters and Cherry[35] measured the effect of protein-to-lipid (P/L) ratio on diffusion. They found that at P/L ratio of 1:140, which is close to the ∼1:150 used in our simulations, lipids diffuse at a rate of 4.3 μ^2/s. This agrees reasonably well with the values of 2–3 μ^2/s calculated for different lipid types in our crowded multi-protein system. In diluted conditions (single-protein case: sections 2.8 and 2.9 in the SI) with a P/L ratio of ∼1:300, extrapolating slightly the data of Peters and Cherry[35], we obtain an estimated lipid diffusion coefficient of ∼10 μ^2/s. This agrees fairly well with the measured lipid diffusion coefficients which lie in the range of 3–5 μ^2/s. The small difference between experimental and calculated diffusion coefficients is due to various reasons. First of all, both the lipid mixture and the proteins used are different. Most notably, our simulations contain cholesterol, which is known to slow down lateral diffusion in lipid membranes. Second, the temperatures employed in their experiment and in our simulations are different. Last, our simulation time is scaled with the suggested Martini force field scaling factor of 4[11]. This naturally results in 4 times smaller diffusion coefficients when compared to simulations where no scaling factor is used. Such scaling factor was suggested based on the dynamics of water and it is worth noting that, despite its widespread use, it might not be the correct factor for other molecule types, especially those larger than water[11].

For proteins in the dilute (single-protein) systems, we measure diffusion coefficients of 0.2–0.5 μ^2/s, which corresponds to non-scaled values of 0.8–2.0 μ^2/s. Diffusion coefficients in the range of 0.1–0.8 μ^2/s were measured for a range of GPCRs in HEK293 cells[36–38]. The oligomerisation tendency of many of these receptors might affect their dynamics in the membrane, yet the effect should only be of the order of 10% according to the Saffman–Delbrück model. Diffusion coefficients of BR, which has a 7-helix structure resembling that of A_{2A} and D_2, were measured in model membranes[35]. Again extrapolating the data to a P/L ratio of ∼1:300, we obtain a value of ∼5 μ^2/s. As for lipids, this is again somewhat larger than our values, which is likely explained by the differences in membrane composition. Another study on BR diffusion in DMPC membranes[39] suggested values of ∼3 μ^2/s. Considering that our membranes contain a higher effective protein concentration, the value is in good qualitative agreement with the diffusion coefficients calculated from our simulation. Finally, drastically smaller values of 0.08 μ^2/s were measured for BR diffusion in penta-monododecylether model membranes[40].

All in all, our diffusion coefficients are in the right ballpark with the experimental ones.

2.18 Effect of DHA on the number of homodimers at equilibrium in living cells

We also measured the effect of DHA on the number of A_{2A}–A_{2A} and D_2–D_2 homodimers in living cells by BRET experiments. As stated in the main manuscript, membrane levels of DHA does not seem to affect the number of A_{2A}–A_{2A} or D_2–D_2 homodimers (see Fig. S23).

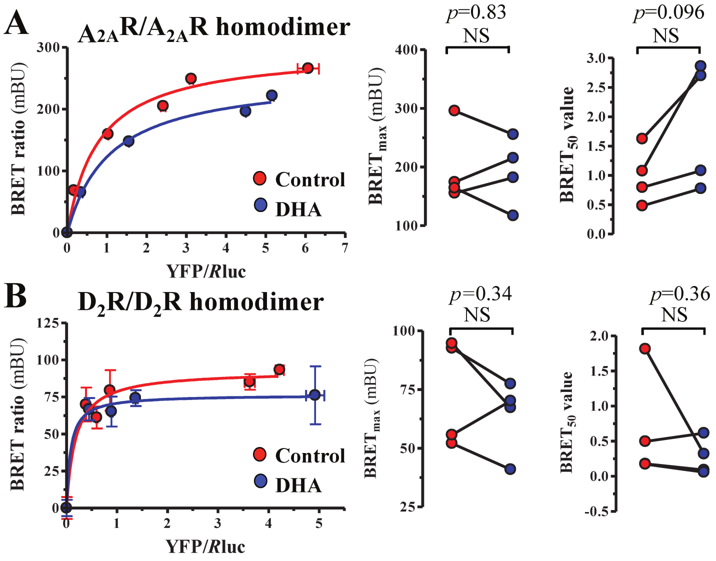

Figure S23: Effect of DHA on the amount of **(A)** A_{2A}–A_{2A} homomerisation, and **(B)** D_2–D_2 homomerisation in cellular steady state with low (red) and high (blue) DHA content. Representative BRET saturation curves are shown in the leftmost column, where each point measurement was performed in triplicate. BRET ratios (x1000) are in mBRET units (mBU). Error bars show the SEM. Middle and rightmost columns show, respectively, the $BRET^{max}$ and $BRET^{50}$ results of 4 independent experiments with low (red) and high (blue) amounts of DHA. These results were compared by a paired t test and the p values are shown

References

[1] V. Jaakola, M. Griffith and M. Hanson, *Science*, 2008, **322**, 1211–1217.

[2] N. Eswar, B. Webb, M. A. Marti-Renom, M. Madhusudhan, D. Eramian, M.-y. Shen, U. Pieper and A. Sali, *Current Protocols in Protein Science*, 2007, **Chapter 2**, Unit 2.9.

[3] L. Monticelli, S. K. Kandasamy, X. Periole, R. G. Larson, D. P. Tieleman and S. J. Marrink, *J. Chem. Theory Comput.*, 2008, **4**, 819–834.

[4] X. Periole, M. Cavalli, S. J. Marrink and M. A. Ceruso, *J. Chem. Theory Comput.*, 2009, **5**, 2531–2543.

[5] R. K. McNamara, C.-G. Hahn, R. Jandacek, T. Rider, P. Tso, K. E. Stanford and N. M. Richtand, *Biol. Psychiat.*, 2007, **62**, 17–24.

[6] R. K. McNamara, R. Jandacek, T. Rider, P. Tso, C.-G. Hahn, N. M. Richtand and K. E. Stanford, *Schizophr. Res.*, 2007, **91**, 37–50.

[7] R. K. McNamara, R. Jandacek, T. Rider, P. Tso, K. E. Stanford, C.-G. Hahn and N. M. Richtand, *Psychiat. Res.*, 2008, **160**, 285–299.

[8] V. Martín, N. Fabelo, G. Santpere, B. Puig, R. Marín, I. Ferrer and M. Díaz, *J. Alzheimers Dis.*, 2010, **19**, 489–502.

[9] N. Fabelo, V. Martín, G. Santpere, R. Marín, L. Torrent, I. Ferrer and M. Díaz, *Mol. Med.*, 2011, **17**, 1107–1118.

[10] A. Y. Taha, Y. Cheon, K. Ma, S. I. Rapoport and J. S. Rao, *J. Psychiat. Res.*, 2013, **47**, 636–643.

[11] S. J. Marrink, H. J. Risselada, S. Yefimov, D. P. Tieleman and A. H. de Vries, *J. Phys. Chem. B*, 2007, **111**, 7812–7824.

[12] M. Javanainen, *J. Chem. Theory Comput.*, 2014, **10**, 2577–2582.

[13] B. Hess and C. Kutzner, *J. Chem. Theory Comput.*, 2008, **4**, 435–447.

[14] H. J. Berendsen, J. P. M. Postma, W. F. van Gunsteren, A. DiNola and J. Haak, *J. Chem. Phys.*, 1984, **81**, 3684–3690.

[15] S. Jo, J. J. B. Lim, J. J. B. Klauda and W. Im, *Biophys. J.*, 2009, **96**, 50–58.

[16] R. M. Venable, A. J. Sodt, B. Rogaski, H. Rui, E. Hatcher, A. D. MacKerell, R. W. Pastor and J. B. Klauda, *Biophys. J.*, 2014, **107**, 134–145.

[17] W. Humphrey, A. Dalke and K. Schulten, *J. Mol. Graphics*, 1996, **14**, 33–38.

[18] K. Vanommeslaeghe and A. D. MacKerell, *J. Chem. Inf. Model*, 2012, **52**, 3144–3154.

[19] K. Vanommeslaeghe, E. P. Raman and A. D. MacKerell, *J. Chem. Inf. Model*, 2012, **52**, 3155–3168.

[20] M. Harvey, G. Giupponi and G. Fabritiis, *J. Chem. Theory Comput.*, 2009, **5**, 1–9.

[21] G. Grest and K. Kremer, *Phys. Rev. A*, 1986, **33**, 3628–3631.

[22] T. Darden, D. York and L. Pedersen, *J. Chem. Phys.*, 1993, **27709**, 13–16.

[23] T. J. Piggot, A. Piñeiro and S. Khalid, *J. Chem. Theory Comput.*, 2012, **8**, 4593âĂŞ–4609.

[24] H. Wickham, *Ggplot2: Elegant Graphics For Data Analysis*, Springer New York, 2009.

[25] G. Lepage and C. C. Roy, *J. Lipid Res.*, 1986, **27**, 114–120.

[26] J. Domański, S. J. Marrink and L. V. Schäfer, *BBA-Biomembranes*, 2012, **1818**, 984–994.

[27] A. Ghosh, U. Sonavane and R. Joshi, *Comput. Biol. Chem.*, 2014, **48**, 29–39.

[28] D. Provasi, M. B. Boz, J. M. Johnston, M. Filizola and A. MacKerell, *PLoS Comp. Biol.*, 2015, **11**, e1004148–e1004148.

[29] X. Periole, A. Knepp, T. P. Sakmar, S. J. Marrink and T. Huber, *J. Am. Chem. Soc.*, 2012, **134**, 10959–10965.

[30] A. C. Stark, C. T. Andrews and A. H. Elcock, *J. Chem. Theory Comput.*, 2013, **9**, 4176–4185.

[31] M. Chavent, A. P. Chetwynd, P. J. Stansfeld and M. S. Sansom, *Biochemistry*, 2014, **53**, 6641–6652.

[32] M. Nishizawa and K. Nishizawa, *J. Chem. Phys.*, 2014, **141**, 075101.

[33] A. May, R. Pool, E. van Dijk, J. Bijlard, S. Abeln, J. Heringa and K. A. Feenstra, *Bioinformatics*, 2014, **30**, 326–334.

[34] D. H. de Jong, G. Singh, W. D. Bennett, C. Arnarez, T. A. Wassenaar, L. V. Schafer, X. Periole, D. P. Tieleman and S. J. Marrink, *J. Chem. Theory Comput.*, 2012, **9**, 687–697.

[35] R. Peters and R. J. Cherry, *P. Natl. Acad. Sci. USA*, 1982, **79**, 4317–4321.

[36] K. Herrick-Davis, E. Grinde, A. Cowan and J. E. Mazurkiewicz, *Mol. pharmacol.*, 2013, **84**, 630–642.

[37] S. Keuerleber, P. Thurner, C. W. Gruber, J. Zezula and M. Freissmuth, *J. of Biol. Chem.*, 2012, **287**, 42104–42118.

[38] A. İ. Kaya, Ö. Uğur, O. Altuntaş, K. Sayar and H. O. Onaran, *BBA-Mol. Cell. Res.*, 2011, **1813**, 1511–1524.

[39] W. L. Vaz, M. Criado, V. M. Madeira, G. Schoellmann and T. M. Jovin, *Biochemistry*, 1982, **21**, 5608–5612.

[40] Y. Gambin, R. Lopez-Esparza, M. Reffay, E. Sierecki, N. Gov, M. Genest, R. Hodges and W. Urbach, *P. Natl. Acad. Sci. USA*, 2006, **103**, 2098–2102.
